# Fine-Scale Mapping at 9p22.2 Identifies Candidate Causal Variants That Modify Ovarian Cancer Risk in *BRCA1* and *BRCA2* Mutation Carriers

**DOI:** 10.1371/journal.pone.0158801

**Published:** 2016-07-27

**Authors:** Elena Vigorito, Karoline B. Kuchenbaecker, Jonathan Beesley, Julian Adlard, Bjarni A. Agnarsson, Irene L. Andrulis, Banu K. Arun, Laure Barjhoux, Muriel Belotti, Javier Benitez, Andreas Berger, Anders Bojesen, Bernardo Bonanni, Carole Brewer, Trinidad Caldes, Maria A. Caligo, Ian Campbell, Salina B. Chan, Kathleen B. M. Claes, David E. Cohn, Jackie Cook, Mary B. Daly, Francesca Damiola, Rosemarie Davidson, Antoine de Pauw, Capucine Delnatte, Orland Diez, Susan M. Domchek, Martine Dumont, Katarzyna Durda, Bernd Dworniczak, Douglas F. Easton, Diana Eccles, Christina Edwinsdotter Ardnor, Ros Eeles, Bent Ejlertsen, Steve Ellis, D. Gareth Evans, Lidia Feliubadalo, Florentia Fostira, William D. Foulkes, Eitan Friedman, Debra Frost, Pragna Gaddam, Patricia A. Ganz, Judy Garber, Vanesa Garcia-Barberan, Marion Gauthier-Villars, Andrea Gehrig, Anne-Marie Gerdes, Sophie Giraud, Andrew K. Godwin, David E. Goldgar, Christopher R. Hake, Thomas V. O. Hansen, Sue Healey, Shirley Hodgson, Frans B. L. Hogervorst, Claude Houdayer, Peter J. Hulick, Evgeny N. Imyanitov, Claudine Isaacs, Louise Izatt, Angel Izquierdo, Lauren Jacobs, Anna Jakubowska, Ramunas Janavicius, Katarzyna Jaworska-Bieniek, Uffe Birk Jensen, Esther M. John, Joseph Vijai, Beth Y. Karlan, Karin Kast, KConFab Investigators, Sofia Khan, Ava Kwong, Yael Laitman, Jenny Lester, Fabienne Lesueur, Annelie Liljegren, Jan Lubinski, Phuong L. Mai, Siranoush Manoukian, Sylvie Mazoyer, Alfons Meindl, Arjen R. Mensenkamp, Marco Montagna, Katherine L. Nathanson, Susan L. Neuhausen, Heli Nevanlinna, Dieter Niederacher, Edith Olah, Olufunmilayo I. Olopade, Kai-ren Ong, Ana Osorio, Sue Kyung Park, Ylva Paulsson-Karlsson, Inge Sokilde Pedersen, Bernard Peissel, Paolo Peterlongo, Georg Pfeiler, Catherine M. Phelan, Marion Piedmonte, Bruce Poppe, Miquel Angel Pujana, Paolo Radice, Gad Rennert, Gustavo C. Rodriguez, Matti A. Rookus, Eric A. Ross, Rita Katharina Schmutzler, Jacques Simard, Christian F. Singer, Thomas P. Slavin, Penny Soucy, Melissa Southey, Doris Steinemann, Dominique Stoppa-Lyonnet, Grzegorz Sukiennicki, Christian Sutter, Csilla I. Szabo, Muy-Kheng Tea, Manuel R. Teixeira, Soo-Hwang Teo, Mary Beth Terry, Mads Thomassen, Maria Grazia Tibiletti, Laima Tihomirova, Silvia Tognazzo, Elizabeth J. van Rensburg, Liliana Varesco, Raymonda Varon-Mateeva, Athanassios Vratimos, Jeffrey N. Weitzel, Lesley McGuffog, Judy Kirk, Amanda Ewart Toland, Ute Hamann, Noralane Lindor, Susan J. Ramus, Mark H. Greene, Fergus J. Couch, Kenneth Offit, Paul D. P. Pharoah, Georgia Chenevix-Trench, Antonis C. Antoniou

**Affiliations:** 1 Centre for Cancer Genetic Epidemiology, Department of Public Health and Primary Care, University of Cambridge, Cambridge, United Kingdom; 2 Department of Genetics, QIMR Berghofer Medical Research Institute, Herston Road, Brisbane, Australia, 4029; 3 Yorkshire Regional Genetics Service, Chapel Allerton Hospital, Leeds, United Kingdom; 4 Department of Pathology, University Hospital (Landspitali) and University of Iceland School of Medicine, Hringbraut, 101 Reykjavik, Iceland; 5 Lunenfeld-Tanenbaum Research Institute, Mount Sinai Hospital, Toronto, Ontario M5G 1X5, Canada; 6 Department of Breast Medical Oncology and Clinical Cancer Genetics Program, University Of Texas MD Anderson Cancer Center, 1515 Pressler Street, CBP 5, Houston, TX, United States of America; 7 Bâtiment Cheney D, Centre Léon Bérard, 28 rue Laënnec, Lyon, France; 8 Service de Génétique Oncologique, Institut Curie, 26, rue d’Ulm, Paris Cedex 05, France; 9 Human Genetics Group, Spanish National Cancer Centre (CNIO), Madrid, Spain, and Human Genotyping (CEGEN) Unit, Human Cancer Genetics Program, Spanish National Cancer Research Centre (CNIO), Madrid, Spain; 10 Dept of OB/GYN, Medical University of Vienna, Vienna, Austria; 11 Department of Clinical Genetics, Vejle Hospital, Kabbeltoft 25, Vejle, Denmark; 12 Division of Cancer Prevention and Genetics, Istituto Europeo di Oncologia (IEO), via Ripamonti 435, 20141 Milan, Italy; 13 Department of Clinical Genetics, Royal Devon & Exeter Hospital, Exeter, United Kingdom; 14 Molecular Oncology Laboratory, Hospital Clinico San Carlos, IdISSC (El Instituto de Investigación Sanitaria del Hospital Clínico San Carlos), Martin Lagos s/n, Madrid, Spain; 15 Section of Genetic Oncology, Dept. of Laboratory Medicine, University and University Hospital of Pisa, Pisa Italy; 16 Research Division, Peter MacCallum Cancer Centre, Locked Bag 1, A'Beckett Street, Melbourne, VIC 8006, Australia; 17 1600 Divisadero Street, C415, San Francisco, CA 94143–1714, United States of America; 18 Center for Medical Genetics, Ghent University, De Pintelaan 185, 9000 Gent, Belgium; 19 Ohio State University Columbus Cancer Council GYN Oncology, 3651 Ridge Mill Drive, Columbus, OH 43026, United States of America; 20 Sheffield Clinical Genetics Service, Sheffield Children’s Hospital, Sheffield, United Kingdom; 21 Department of Clinical Oncology, Fox Chase Cancer Center, 333 Cottman Avenue, Philadelphia, PA, United States of America; 22 Department of Clinical Genetics, South Glasgow University Hospitals, Glasgow, United Kingdom; 23 Unité d'oncogénétique, ICO-Centre René Gauducheau, Boulevard Jacques Monod, 44805 Nantes Saint Herblain Cedex, France; 24 Oncogenetics Group, Vall d’Hebron University Hospital, Vall d’Hebron Institute of Oncology (VHIO), and Universitat Autònoma, Passeig Vall d'Hebron 119–129, Barcelona, Spain; 25 Department of Medicine, Abramson Cancer Center, Perelman School of Medicine at the University of Pennsylvania, 3400 Civic Center Boulevard, Philadelphia, PA 19104, United States of America; 26 Genomics Center, Centre Hospitalier Universitaire de Québec Research Center and Laval University, 2705 Laurier Boulevard, Quebec City (Quebec), Canada; 27 Department of Genetics and Pathology, Pomeranian Medical University, Polabska 4, Szczecin, Poland; 28 Institute of Human Genetics, University of Münster, Münster, Germany; 29 University of Southampton Faculty of Medicine, Southampton University Hospitals NHS Trust, Southampton, United Kingdom; 30 Department of Radiation Sciences, Oncology, Umeå University, Umea, Sweden; 31 Oncogenetics Team, The Institute of Cancer Research and Royal Marsden NHS Foundation Trust, Sutton, United Kingdom; 32 Department of Oncology, Rigshospitalet, Copenhagen University Hospital, Blegdamsvej 9, DK-2100 Copenhagen, Denmark; 33 Centre for Cancer Genetic Epidemiology, Department of Public Health and Primary Care, University of Cambridge, Strangeways Research Laboratory, Worts Causeway, Cambridge, United Kingdom; 34 Genomic Medicine, Manchester Academic Health Sciences Centre, Institute of Human Development, Manchester University, Central Manchester University Hospitals NHS Foundation Trust, Manchester, United Kingdom; 35 Molecular Diagnostic Unit, Hereditary Cancer Program, IDIBELL (Bellvitge Biomedical Research Institute), Catalan Institute of Oncology, Gran Via de l'Hospitalet, 199–203, 08908 L'Hospitalet, Barcelona, Spain; 36 Molecular Diagnostics Laboratory, (INRASTES) Institute of Nuclear and Radiological Sciences and Technology, National Centre for Scientific Research "Demokritos", Patriarchou Gregoriou & Neapoleos str., Aghia Paraskevi, Attiki, Athens, Greece; 37 Program in Cancer Genetics, Departments of Human Genetics and Oncology, McGill University, Montreal, Quebec, Canada; 38 The Susanne Levy Gertner Oncogenetics Unit, Institute of Human Genetics, Chaim Sheba Medical Center, Ramat Gan 52621, and Sackler Faculty of Medicine, Tel Aviv University, Ramat Aviv 69978, Israel; 39 Clinical Cancer Genetics Laboratory, Memorial Sloane Kettering Cancer Center, New York, NY, United States of America; 40 UCLA Schools of Medicine and Public Health, Division of Cancer Prevention & Control Research, Jonsson Comprehensive Cancer Center, 650 Charles Young Drive South, Room A2-125 HS, Los Angeles, CA 90095–6900, United States of America; 41 Cancer Risk and Prevention Clinic, Dana-Farber Cancer Institute, 450 Brookline Avenue, Boston, MA, United States of America; 42 Centre of Familial Breast and Ovarian Cancer, Department of Medical Genetics, Institute of Human Genetics, University Würzburg, Würzburg, Germany; 43 Department of Clinical Genetics, Rigshospitalet 4062, Blegdamsvej 9, København Ø, Denmark; 44 Service de Génétique Moléculaire et Clinique, Hospices Civils de Lyon, Lyon cedex 04, France; 45 Department of Pathology and Laboratory Medicine, 3901 Rainbow Boulevard, 4019 Wahl Hall East, MS 3040, University of Kansas Medical Center, Kansas City, Kansas, United States of America; 46 Department of Dermatology, University of Utah School of Medicine, 30 North 1900 East, SOM 4B454, Salt Lake City, UT 84132, United States of America; 47 City of Hope Clinical Cancer Genetics Community Research Network, 1500 East Duarte Road, Duarte, CA 91010, United States of America; 48 Center for Genomic Medicine, Rigshospitalet, Copenhagen University Hospital, Blegdamsvej 9, DK-2100 Copenhagen, Denmark; 49 Medical Genetics Unit, St George's, University of London, London, United Kingdom; 50 Family Cancer Clinic, Netherlands Cancer Institute, P.O. Box 90203, 1000 BE, Amsterdam, The Netherlands; 51 Center for Medical Genetics, NorthShore University Health System, University of Chicago Pritzker School of Medicine, 1000 Central Street, Suite 620, Evanston, IL 60201, United States of America; 52 N.N. Petrov Institute of Oncology, St.-Petersburg 197758, Russia; 53 Lombardi Comprehensive Cancer Center, Georgetown University, 3800 Reservoir Road NW, Washington, DC, United States of America; 54 Clinical Genetics, Guy’s and St. Thomas’ NHS Foundation Trust, London, United Kingdom; 55 Genetic Counseling Unit, Hereditary Cancer Program, IDIBGI (Institut d'Investigació Biomèdica de Girona), Catalan Institute of Oncology, Av. França s/n. 1707 Girona, Spain; 56 Clinical Genetics Research Laboratory, Dept. of Medicine, Memorial Sloan-Kettering Cancer Center, 1275 York Avenue, New York, NY, United States of America; 57 Vilnius University Hospital Santariskiu Clinics, Hematology, oncology and transfusion medicine center, Dept. of Molecular and Regenerative Medicine, Santariskiu st., State Research Institute Centre for Innovative medicine, Zygymantu st. 9, Vilnius, Lithuania; 58 Department of Clinical Genetics, Aarhus University Hospital, Brendstrupgaardsvej 21C, Aarhus N, Denmark; 59 Department of Epidemiology, Cancer Prevention Institute of California, 2201 Walnut Avenue, Suite 300, Fremont, CA 94538, United States of America; 60 Clinical Genetics Research Laboratory, Dept. of Medicine, Memorial Sloan-Kettering Cancer Center, 1275 York Avenue, New York, NY 10044, United States of America; 61 Women's Cancer Program at the Samuel Oschin Comprehensive Cancer Institute, Cedars-Sinai Medical Center, 8700 Beverly Boulevard, Suite 290W, Los Angeles, CA, United States of America; 62 Department of Gynaecology and Obstetrics, University Hospital Carl Gustav Carus, Technical University Dresden, Dresden, Germany; 63 Kathleen Cuningham Consortium for Research into Familial Breast Cancer, Peter MacCallum Cancer Center, Melbourne, Australia; 64 Department of Obstetrics and Gynecology, University of Helsinki and Helsinki University Hospital, Biomedicum Helsinki, P.O. BOX 700 (Haartmaninkatu 8), 00029 HUS, Finland; 65 The Hong Kong Hereditary Breast Cancer Family Registry, Cancer Genetics Center, Hong Kong Sanatorium and Hospital, Hong Kong China; 66 The Susanne Levy Gertner Oncogenetics Unit, Institute of Human Genetics, Chaim Sheba Medical Center, Ramat Gan 52621, Israel; 67 Genetic Epidemiology of Cancer team, Inserm U900, Institut Curie, Mines ParisTech, 26 rue d'Ulm, 75248 Paris cedex 05, France; 68 Department of Oncology, Karolinska University Hospital, Stockholm, Sweden; 69 Clinical Genetics Branch, DCEG, NCI, NIH, 9609 Medical Center Drive, Room 6E-454, Bethesda, MD, United States of America; 70 Unit of Medical Genetics, Department of Preventive and Predictive Medicine, Fondazione IRCCS (Istituto Di Ricovero e Cura a Carattere Scientifico) Istituto Nazionale Tumori (INT), Via Giacomo Venezian 1, 20133 Milan, Italy; 71 Department of Gynaecology and Obstetrics, Division of Tumor Genetics, Klinikum rechts der Isar, Technical University Munich, Munich, Germany; 72 Department of Human Genetics, Radboud university medical centre, P.O. Box 9101, 6500 HB Nijmegen, The Netherlands; 73 Immunology and Molecular Oncology Unit, Veneto Institute of Oncology IOV—IRCCS, Via Gattamelata 64, Padua, Italy; 74 Department of Population Sciences, Beckman Research Institute of City of Hope, Duarte, CA, United States of America; 75 Department of Gynaecology and Obstetrics, University Hospital Düsseldorf, Heinrich-Heine University Düsseldorf, Düsseldorf, Germany; 76 Department of Molecular Genetics, National Institute of Oncology, Budapest, Hungary; 77 The University of Chicago Medicine, 5841 South Maryland Avenue, MC 2115 Chicago, IL 60637; 78 West Midlands Regional Genetics Service, Birmingham Women’s Hospital Healthcare NHS Trust, Edgbaston, Birmingham, United Kingdom; 79 Human Genetics Group, Spanish National Cancer Centre (CNIO), Madrid, Spain; 80 Department of Preventive Medicine, Seoul National University College of Medicine, Department of Biomedical Science, Seoul National University Graduate School, and Cancer Research Institute, Seoul National University, 103 Daehak-ro, Jongno-gu, Seoul 110–799, Korea; 81 Department of Immunology, Genetics and Pathology, Uppsala University, SE-751 85 Uppsala, Sweden; 82 Section of Molecular Diagnostics, Department of Biochemistry, Aalborg University Hospital, Reberbansgade 15, Aalborg, Denmark; 83 IFOM, The FIRC (Italian Foundation for Cancer Research) Institute of Molecular Oncology, c/o IFOM-IEO campus, via Adamello 16, 20139 Milan, Italy; 84 Medical University of Vienna, Währinger Gürtel 18–20, 1090 Vienna, Austria; 85 Department of Cancer Epidemiology, Moffitt Cancer Center, Tampa, Florida, United States of America; 86 NRG Oncology, Statistics and Data Management Center, Roswell Park Cancer Institute, Elm St & Carlton St, Buffalo, NY 14263, United States of America; 87 Translational Research Laboratory, IDIBELL (Bellvitge Biomedical Research Institute), Catalan Institute of Oncology, Barcelona, Spain; 88 Unit of Molecular Bases of Genetic Risk and Genetic Testing, Department of Preventive and Predicted Medicine, Fondazione IRCCS (Istituto Di Ricovero e Cura a Carattere Scientifico) Istituto Nazionale Tumori (INT), c/o Amaedeolab, via GA Amadeo 42, 20133 Milan, Italy; 89 Clalit National Israeli Cancer Control Center and Department of Community Medicine and Epidemiology, Carmel Medical Center and B. Rappaport Faculty of Medicine, 7 Michal St., Haifa 34362, Israel; 90 Division of Gynecologic Oncology, NorthShore University HealthSystem, Univ of Chicago, 2650 Ridge Avenue Suite 1507 Walgreens, Evanston, IL 60201, United States of America; 91 Department of Epidemiology, Netherlands Cancer Institute, P.O. Box 90203, 1000 BE, Amsterdam, The Netherlands; 92 Biostatistics and Bioinformatics Facility, Fox Chase Cancer Center, 333 Cottman Avenue, Philadelphia, PA 19111, United States of America; 93 Center for Hereditary Breast and Ovarian Cancer, Medical Faculty, University Hospital Cologne, Cologne, Germany; 94 Dept of OB/GYN, Medical University of Vienna, Vienna, Austria, and Waehringer Guertel 18–20, A 1090 Vienna, Austria; 95 Clinical Cancer Genetics, City of Hope, 1500 East Duarte Road, Duarte, California 91010, United States of America; 96 Genetic Epidemiology Laboratory, Department of Pathology, University of Melbourne, Parkville, Victoria, Australia; 97 Institute of Cell and Molecular Pathology, Hannover Medical School, Hannover, Germany; 98 Institute of Human Genetics, Department of Human Genetics, University Hospital Heidelberg, Heidelberg, Germany; 99 National Human Genome Research Institute, National Institutes of Health, Building 50, Room 5312, 50 South Drive, MSC 004, Bethesda, MD 20892–8004, United States of America; 100 Department of Genetics, Portuguese Oncology Institute, Rua Dr. António Bernardino de Almeida, 4200–072 Porto, Portugal; 101 Cancer Research Initiatives Foundation, Sime Darby Medical Centre, 1 Jalan SS12/1A, Subang Jaya, 47500 Malaysia; 102 Department of Epidemiology, Columbia University, New York, NY, United States of America; 103 Department of Clinical Genetics, Odense University Hospital, Sonder Boulevard 29, Odense C, Denmark; 104 UO Anatomia Patologica, Ospedale di Circolo-Università dell'Insubria, Via O.Rossi 9, 21100 Varese, Italy; 105 Latvian Biomedical Research and Study Centre, Ratsupites str 1, Riga, Latvia; 106 Cancer Genetics Laboratory, Department of Genetics, University of Pretoria, Private Bag X323, Arcadia 0007, South Africa; 107 Unit of Hereditary Cancer, Department of Epidemiology, Prevention and Special Functions, IRCCS (Istituto Di Ricovero e Cura a Carattere Scientifico) AOU San Martino—IST Istituto Nazionale per la Ricerca sul Cancro, largo Rosanna Benzi 10, 16132 Genoa, Italy; 108 Institute of Human Genetics, Campus Virchov Klinikum, Charite Berlin, Berlin, Germany; 109 Molecular Diagnostics Laboratory, (INRASTES) Institute of Nuclear and Radiological Sciences and Technology, National Centre for Scientific Research "Demokritos" Patriarchou Gregoriou & Neapoleos str., Aghia Paraskevi, Attiki, Athens, Greece; 110 Westmead Hospital, Familial Cancer Service, Hawkesbury Road, P.O. Box 533, Wentworthville, NSW 2145, Australia; 111 Divison of Human Cancer Genetics, Departments of Internal Medicine and Molecular Virology, Immunology and Medical Genetics, Comprehensive Cancer Center, The Ohio State University, 998 Biomedical Research Tower, Columbus, OH, United States of America; 112 Molecular Genetics of Breast Cancer, German Cancer Research Center (DKFZ), Im Neuenheimer Feld 580, 69120 Heidelberg, Germany; 113 Department of Health Sciences Research, Mayo Clinic, 13400 E. Scottsdale Blvd., Scottsdale, AZ, United States of America; 114 Department of Preventive Medicine, Keck School of Medicine, University of Southern California, California, United States of America; 115 Department of Laboratory Medicine and Pathology, and Health Sciences Research, Mayo Clinic, 200 First Street SW, Rochester, Minnesota, United States of America; 116 Clinical Genetics Research Laboratory, Dept. of Medicine, Cancer Biology and Genetics, Memorial Sloan-Kettering Cancer Center, 1275 York Avenue, New York, NY 10044, United States of America; 117 Department of Oncology, University of Cambridge, Cambridge, United Kingdom; National Cancer Institute, National Institutes of Health, UNITED STATES

## Abstract

Population-based genome wide association studies have identified a locus at 9p22.2 associated with ovarian cancer risk, which also modifies ovarian cancer risk in *BRCA1* and *BRCA2* mutation carriers. We conducted fine-scale mapping at 9p22.2 to identify potential causal variants in *BRCA1* and *BRCA2* mutation carriers. Genotype data were available for 15,252 (2,462 ovarian cancer cases) *BRCA1* and 8,211 (631 ovarian cancer cases) *BRCA2* mutation carriers. Following genotype imputation, ovarian cancer associations were assessed for 4,873 and 5,020 SNPs in *BRCA*1 and *BRCA* 2 mutation carriers respectively, within a retrospective cohort analytical framework. In *BRCA1* mutation carriers one set of eight correlated candidate causal variants for ovarian cancer risk modification was identified (top SNP rs10124837, HR: 0.73, 95%CI: 0.68 to 0.79, p-value 2× 10−16). These variants were located up to 20 kb upstream of *BNC2*. In *BRCA2* mutation carriers one region, up to 45 kb upstream of *BNC2*, and containing 100 correlated SNPs was identified as candidate causal (top SNP rs62543585, HR: 0.69, 95%CI: 0.59 to 0.80, p-value 1.0 × 10−6). The candidate causal in *BRCA1* mutation carriers did not include the strongest associated variant at this locus in the general population. In sum, we identified a set of candidate causal variants in a region that encompasses the *BNC2* transcription start site. The ovarian cancer association at 9p22.2 may be mediated by different variants in *BRCA1* mutation carriers and in the general population. Thus, potentially different mechanisms may underlie ovarian cancer risk for mutation carriers and the general population.

## Introduction

Once age is taken into account, family history is the strongest risk factor for ovarian cancer. Women with a first-degree relative with ovarian cancer are at a 3-fold increased risk of developing the disease, indicating the importance of genetic factors in ovarian cancer predisposition. The most important genes in the context of genetic counseling for ovarian cancer susceptibility are *BRCA1* and *BRCA2*, which account for approximately 24% of the familial risk among first-degree relatives [1]. In contrast to the general population, in which the lifetime risk of developing ovarian carcinoma is 1.6% (average age at diagnosis 63 years), women carrying a *BRCA1* mutation have a lifetime risk of 35–60% with an average age of diagnosis of 50 years [2]. The ovarian cancer penetrance is lower for *BRCA2*, with a lifetime risk of 12–25% and an average age of diagnosis of 60 years [2]. The majority of *BRCA1/2* associated ovarian cancers present as high-grade serous histology in advanced stage [3].

Genome wide association studies have identified several common germline variants associated with ovarian cancer risk. The 9p22 locus was first found to be associated with ovarian cancer risk in the general population, and subsequently to be an ovarian cancer risk modifier in *BRCA1* and *BRCA2* mutation carriers [4,5]. The SNP showing the strongest association in the general population was rs3814113, which was associated with a decrease in the risk of ovarian cancer in carriers of the minor allele (OR per allele = 0.82, 95%CI: 0.79 to 0.86, p-value = 5.1 × 10−19) [5] and had a similar association with ovarian cancer risk for *BRCA1* and *BRCA2* mutation carriers [4]. rs3814113 lies in a 150-kb linkage disequilibrium (LD) block. The closest genes to rs3814113 are *Basonuclin 2(BNC2)* and *Centlein (CNTLN)*. *BNC2* is a zinc-finger protein spanning nucleotides 16409503 to 16870706. It is expressed in ovary, testis and the male germ line where it regulates cell cycle progression [6]. *CNTLN* spans nucleotides 17134982 to 17503923, it is ubiquitously expressed and localises at centrosomes to ensure centrosome function during cell division [7,8]. However, no fine-scale mapping of this locus has been reported yet in either the general population or in mutation carriers. Therefore, it is unclear which are the likely causal variants in the region.

Here, we report the fine-scale mapping of the 9p22.2 locus using data from 15252 *BRCA1* and 8211 *BRCA2* mutation carriers of European ancestry. We comprehensively characterized the associations of genetic variants in the region with ovarian cancer risk for *BRCA1* and *BRCA2* mutation carriers.

## Materials and Methods

### Study Population

Epidemiological and genotype data were obtained from *BRCA1* and *BRCA2* mutation carriers participating in the Consortium of Investigators of Modifiers of *BRCA1/2* (CIMBA, [9]). Eligibility to CIMBA was restricted to women older than age 18 years who carried pathogenic mutations in the *BRCA1* or *BRCA2* genes. For each mutation carrier, date of birth, age at cancer diagnosis, age at bilateral prophylactic mastectomy and/or oophorectomy, age at interview or last follow-up, exact *BRCA1* and *BRCA2* mutation description and self-reported ethnicity were recorded, together with tumor pathology, survival, treatment and other established lifestyle/hormonal risk factors for breast or ovarian cancer. Participants were recruited from 25 countries under ethically approved protocols and provided written informed consent.

### Genotyping and Imputation

Genotyping was performed using the iCOGS Illumina array [10]. The quality control (QC) of the genotyping data has been described in detail previously [11,12]. The iCOGS array included SNPs for fine mapping of the 9p22.2 region. The fine mapping region was defined as Chromosome 9 positions: 16407967 to 17407967 (NCBI build 37). To select the SNPs for inclusion on iCOGS, we considered all variants with minor allele frequencies of >0.02 from the 1000 Genomes Project (March 2010 version) and selected SNPs that were correlated at r^2^>0.1 with the SNP that had been identified through the GWAS (rs3814113), and the set of SNPs that tagged all remaining SNPs in the region with r^2^>0.9. A total of 407 and 401 SNPs that were included on iCOGS in the 9p22.2 region passed QC and were available for the analyses for *BRCA1* and *BRCA2* mutation carriers, respectively. Imputation of genotypes was based on the phase 3 release of the 1000 Genome Project spanning nucleotides 16407967 to 17407967 (build 37) at chromosome 9 with a buffer region of 500bp, using IMPUTE2 v2 [13]. SNPs with an “info” metric lower than 0.3 were considered poorly imputed and excluded from downstream analyses. In addition, SNPs with a minor allele frequency (MAF) lower than 0.005 were excluded from the association analyses.

### Statistical Analysis and Computational Methods

The primary analysis evaluated the association between each variant and ovarian cancer risk. To account for the non-random sampling of mutation carriers with respect to disease status, the analysis was conducted within a retrospective cohort framework by modeling the likelihood of the observed genotypes conditional on the disease phenotypes as previously described [14]. Each mutation carrier was followed until the first of: ovarian cancer diagnosis, risk-reducing salpingo-oophorectomy or age at last observation. Only those diagnosed with ovarian cancer were considered as cases. The effect of each SNP was modeled as a per-allele Hazard Ratio (HR). To account for related individuals in the sample, a kinship-adjusted version of the score test for association was used which accounts for the correlation between the genotypes of the relatives [15]. Analyses were carried out separately for *BRCA1* and *BRCA2* mutation carriers and all analyses were stratified by country of residence and year of birth. The USA and Canada strata were further subdivided by reported Ashkenazi Jewish ancestry.

Ovarian cancer associations were combined in a meta-analysis between *BRCA1* and *BRCA2* mutation carriers. A fixed effect meta-analysis weighted by the inverse variance was conducted for imputed and genotyped SNPs when risk estimates were available in both datasets. For *BRCA1* and *BRCA2* mutation carriers, logarithms of per-allele HR estimates were used. The Cochran Q test was carried out to assess heterogeneity.

To assess the number of variants independently associated with ovarian cancer risk in *BRCA1* and *BRCA2* mutation carriers, each SNP was included in a Cox-regression model conditioned on the most strongly associated variant for each dataset and further adjusting by year of birth, and stratifying by country of residence. This approach has been shown to yield valid tests of association [16]. All SNPs with a MAF>0.005, and imputation accuracy higher than 0.3, were included. For single SNP associations, associations were considered significant if p<5x10^-8^. The most parsimonious model in the conditional analyses was identified using a threshold of p<10^−4^ for retaining SNPs in the model.

The set of potential causal SNPs was defined by those SNPs for which their likelihood ratio relative to the most significant variant was equal or less than 100 and having a pair-wise correlation (*r*2) with the top SNP higher than 0.1 [17].

BEDTools was used to intersect positions of ovarian cancer risk-associated variants with functional genomic features generated by Coetzee et al [18] including FAIRE-seq identified regulatory elements and enhancers identified by histone modification ChIP-seq. Variants implicated by overlap were then queried with HaploReg v3 (http://www.broadinstitute.org/mammals/haploreg/haploreg_v3.php).

### Ethics statement

Each of the host institutions recruited under ethically approved protocols. A list of the local Institutional Review Boards that provided ethical approval for this study is given in S1 Table.

## Results

### Association of the 9p22.2 Locus with Ovarian Cancer Risk in *BRCA1* Mutation Carriers

Data were available for 15,252 *BRCA1* mutation carriers of whom 2,462 were censored at ovarian cancer diagnosis (S2 Table). After quality control, data for 407 SNPs genotyped through the iCOGS array spanning chromosome 9 from positions 16424985 to 174 04464 (Genome built 37) were available. A further 36,769 SNPs were imputed using the 1000 Genome Project as reference panel. Of those, 4,873 had a MAF higher than 0.005 and were considered reliably imputed (IMPUTE2 "info" score > 0.3), and were included in the association analysis.

**Fig 1:**
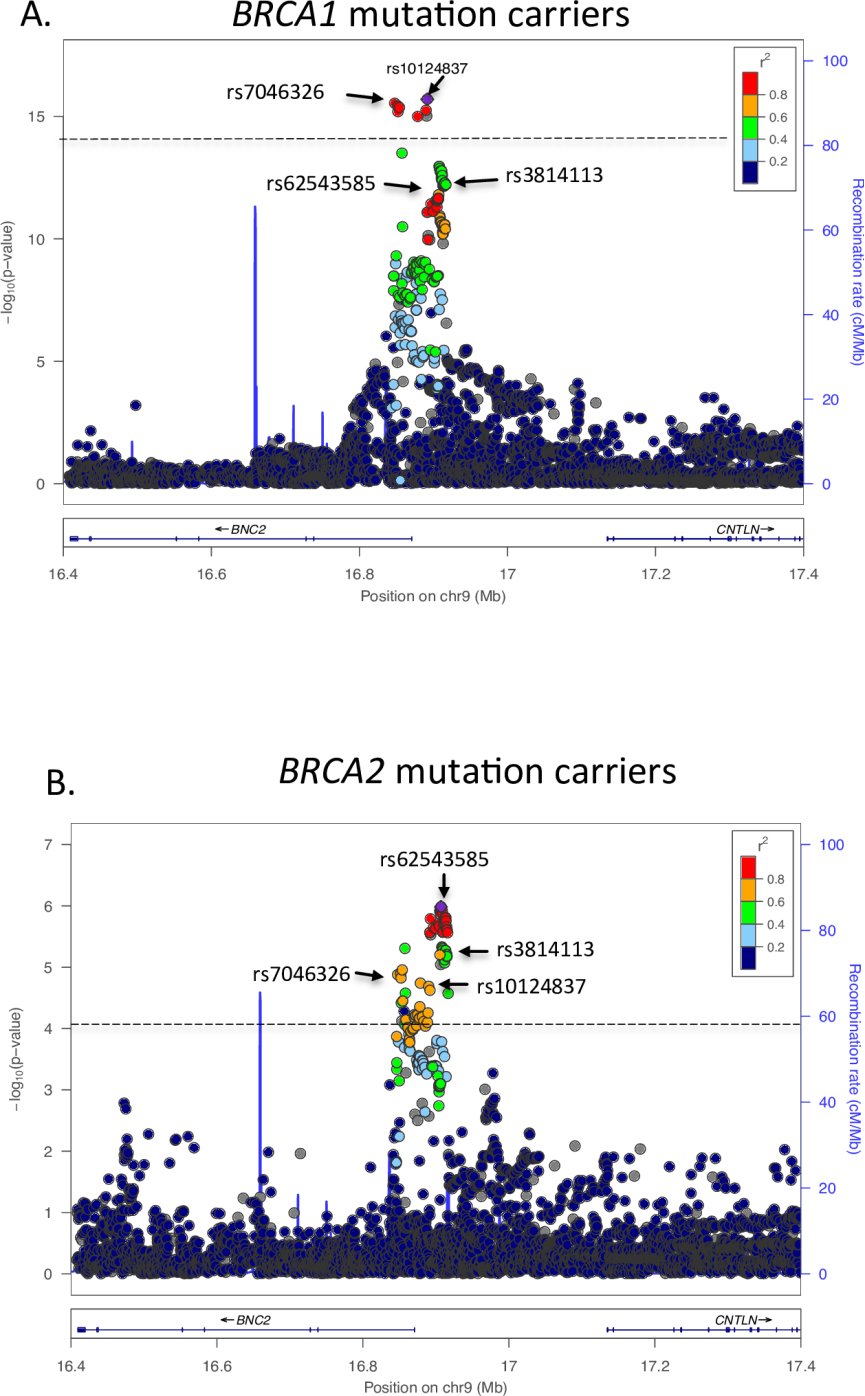
Associations between SNPs in 9p22.2 with ovarian cancer risk for *BRCA1* and *BRCA2* mutation carriers. In each plot, the purple diamond corresponds to the strongest associated SNP and the colour code indicates the linkage disequilibrium with respect to this variant. Horizontal lines indicate the -log_10_ p-value such that the SNPs above the line are the potential causal ones. This set was defined based on a likelihood ratio for a particular SNP as being less or equal than 100, relative to the most likely variant and r^2^>0.1. (A) *BRCA1* mutation carriers, (B) *BRCA2* mutation carriers.

The strongest associated variant was the imputed SNP rs10124837 (per allele HR = 0.73; 95%CI = 0.68–0.79; p = 2.0×10−16, Fig 1A, Table 1 and S1 Table) located 12 kb upstream of *BNC2*. SNP rs3814113 that was originally identified through the GWAS demonstrated a weaker association (p = 5.2x10^-13^). The correlation between the top SNP and the rs3814113 was 0.56 (Table 2). In total, 292 SNPs showed evidence of association with ovarian cancer risk (p: 10^−4^ to 10^−16^, Fig 1A). The correlation between the top SNP and the SNPs in this set varied from 0.1 to 0.9 (Fig 1). Results for all SNPs are presented in S3 Table.

**Table 1:** Associations between selected SNPs from 9p22.2 and ovarian cancer in *BRCA1*, *BRCA2* and combined analysis of *BRCA1/2* mutation carriers.

					*BNC2*	*BRCA1* (all 15252, affected 2462)					*BRCA2* (all 8211, affected 631)						*BRCA1/2* meta-analysis		
SNP	Position	R	E.	T	eSNP(p)	Info	MAF	HR	95%CI	p-value	Info	MAF	HR	95%CI	p-value	p-het	HR	95%CI	p-value
**rs10124837**	16891647	T	C	N	4.1E-06	0.98	0.24	0.73	(0.79,0.68)	**2.0E-16**	0.98	0.23	0.74	(0.85,0.64)	2.4E-05	0.90	0.73	(0.69,0.78)	7.5E-21
**rs7046326**	16847520	G	A	Y	6.8E-06	0.99	0.25	0.74	(0.69,0.79)	2.9E-16	1	0.24	0.74	(0.64,0.84)	1.3E-05	0.90	0.74	(0.69,0.79)	**6.2E-21**
rs4961501	16851678	G	T	N	NA	0.97	0.25	0.74	(0.79,0.69)	3.8E-16	0.98	0.24	0.74	(0.84,0.64)	1.3E-05	1.00	0.74	(0.69,0.79)	7.8E-21
rs10810647	16853779	T	C	N	NA	0.98	0.25	0.74	(0.79,0.69)	4.4E-16	0.98	0.24	0.73	(0.84,0.64)	1.1E-05	0.90	0.74	(0.69,0.79)	7.9E-21
rs10962662	16889937	C	A	Y	1.9E-06	1	0.24	0.74	(0.68,0.79)	5.7E-16	1	0.23	0.74	(0.64,0.85)	2.1E-05	0.90	0.74	(0.69,0.79)	1.9E-20
rs7868157	16851977	A	C	N	NA	0.97	0.24	0.74	(0.79,0.69)	6.5E-16	0.94	0.24	0.74	(0.85,0.64)	1.5E-05	1.00	0.74	(0.69,0.79)	1.6E-20
rs139555631	16890684	C	CTATT	N	NA	0.9	0.28	0.74	(0.79,0.68)	9.7E-16	0.9	0.27	0.77	(0.88,0.67)	2.4E-04	0.54	0.74	(0.7,0.79)	4.1E-19
rs10756823	16878616	C	A	N	2E-07	0.98	0.24	0.74	(0.69,0.79)	1.0E-15	0.98	0.23	0.74	(0.64,0.85)	1.8E-05	0.90	0.74	(0.69,0.79)	3.1E-20
**rs62543585**	16906889	T	C	Y	NA	1	0.2	0.75	(0.69,0.81)	1.6E-12	1	0.19	0.69	(0.59,0.80)	**1.0E-06**	0.55	0.72	(0.67,0.77)	1.6E-17
**rs3814113**	16915021	T	C	Y	3.7E-07	1	0.33	0.78	(0.73,0.83)	5.2E-13	1	0.32	0.75	(0.66,0.85)	6.7E-06	0.37	0.76	(0.73,0.83)	7.5E-18

Selected SNPs correspond to the 8 strongest associated in *BRCA1* mutation carriers plus the strongest associated SNP in *BRCA2* mutation carriers and the initial GWAS hit rs3814113. SNPs indicated in bold indicate the strongest associated in *BRCA1* mutation carriers, the strongest associated in the *BRCA1/2* meta-analysis, in *BRCA2* mutation carriers and rs3814113. “R” and “E” correspond to reference and effector allele, respectively. “T” corresponds to genotyped, eSNP(p) displays the p-value for expressed Single Nucleotide Polymorphism association for the *BNC2* gene based on whole blood tissue extracted from GTEx Portal (http://www.gtexportal.org/home/). “Info” quantifies the accuracy of the imputation. “MAF”, “HR” and “CI” correspond to minor allele frequency, hazard ration and confidence interval, respectively. P-Het corresponds to the p-value for testing heterogeneity between *BRCA1* and *BRCA2* coefficients of association.

**Table 2:** Pairwise correlations (r^2^) between selected SNPs. SNPs correspond to: rs10124837, the strongest associated in *BRCA1*; rs62543583, the strongest associated in *BRCA2* mutation carriers; rs7046326, the strongest associated in *BRCA1/2* meta-analysis; rs3814113, was the strongest associated variant in the initial GWAS analysis.

SNP	rs10124837	rs62543583	rs7046326	rs3814113
rs10124837	1	0.76	0.88	0.56
rs62543583	0.76	1	0.69	0.48
rs7046326	0.88	0.69	1	0.49
rs3814113	0.56	0.48	0.49	1

### Association of the 9p22.2 Locus with Ovarian Cancer Risk in *BRCA2* Mutation Carriers

A total of 8,211 *BRCA2* mutation carriers were included in the analysis, of whom 631 were censored at ovarian cancer diagnosis (S2 Table). The association analysis included 5,020 SNPs (401 genotyped) with MAF>0.005 that were reliably imputed (IMPUTE2 "info" score greater than 0.3). The strongest associated SNP with ovarian cancer risk was rs62543585, with a MAF of 0.20 and a per-allele HR = 0.69 (95%CI = 0.59–0.80; p = 1.0 × 10−6, Table 1). SNP rs3814113 demonstrated a slightly weaker association (p = 6.7x10^-6^ Table 1, r^2^ with SNP rs62543583 = 0.48, Table 2). Although for BRCA2 mutation carriers the p-values did not reach GWAS statistical significance (5x10^-8^), given the strong prior evidence of association between SNPs in the region and risk for *BRCA1* carriers and in the general population we selected the most significant SNPs as associated with ovarian cancer risk. Results for all SNPs with p<0.01 are presented in S3 Table.

### Meta-analysis of *BRCA1* and *BRCA2* Mutation Carriers

**Fig 2:**
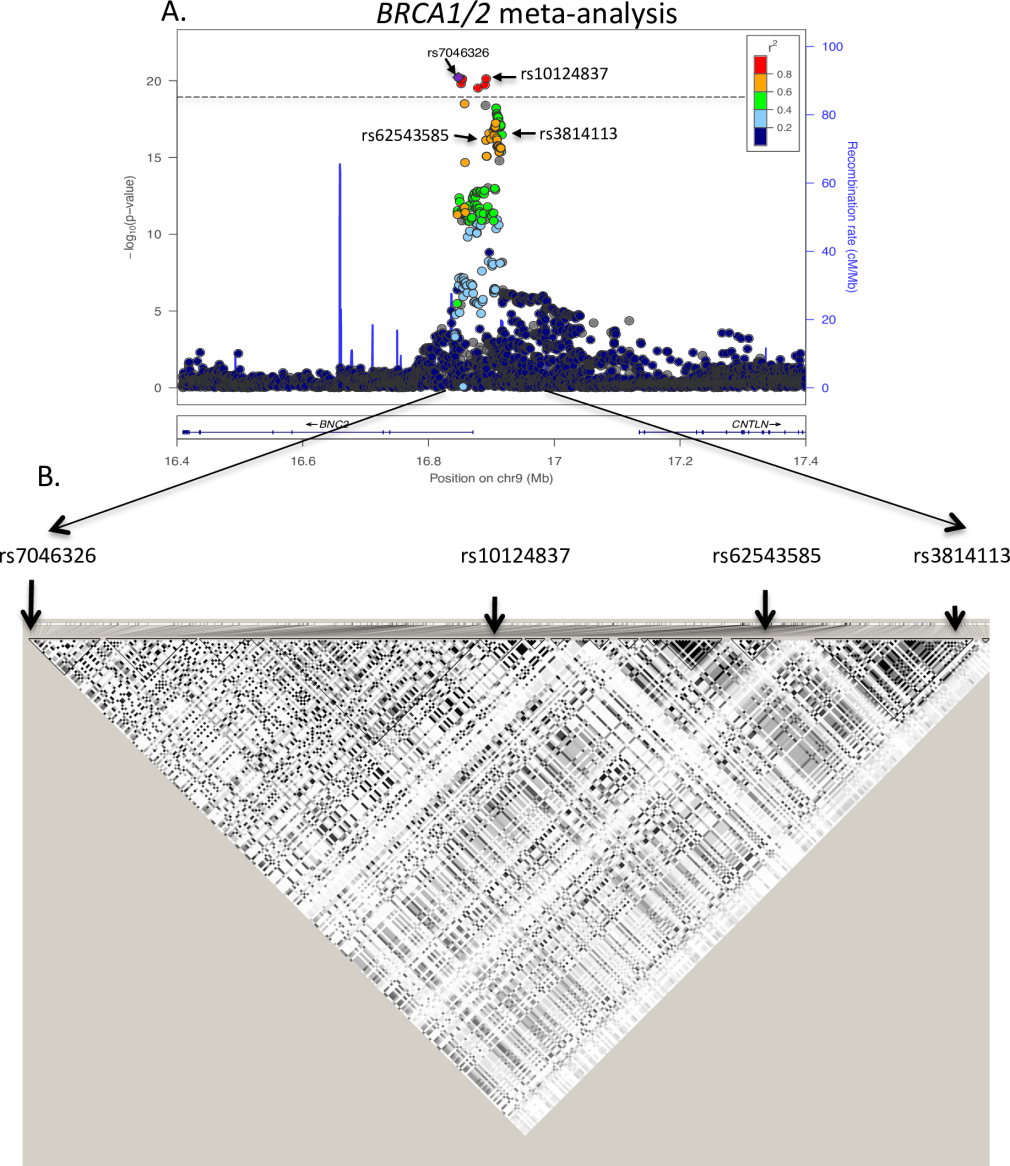
Associations between SNPs in 9p22.2 with ovarian cancer risk for the meta-analysis of *BRCA1* and *BRCA2* mutation carriers. (A) The purple diamond corresponds to the strongest associated SNP and the colour code indicates the linkage disequilibrium with respect to this variant. Horizontal lines indicate the -log_10_ p-value such that the SNPs above the line are the potential causal ones. This set was defined based on a likelihood ratio for a particular SNP as being less or equal than 100, relative to the most likely variant and r^2^>0.1. (B) Haplotype block indicating relevant SNPs. From left to right the indicated SNPs correspond to: the strongest associated in *BRCA1/2* meta-analysis, the strongest in *BRCA1* and the strongest in *BRCA2*.

Since the majority of both *BRCA1* and *BRCA2* ovarian cancer associated cancer tumors are high-grade serous ([19] and S2 Table) to increase the power of the association analyses, a meta-analysis combining HRs for the association of variants with ovarian cancer risk in *BRCA1* and *BRCA2* was conducted. Variants available in only one of the datasets were excluded from the analysis (40 removed from *BRCA1* and 187 from *BRCA2*). In the meta-analysis, the strongest associated variant was the genotyped SNP rs7046326 with a MAF of 0.25 and 0.24 in *BRCA1* and *BRCA2* mutation carriers, respectively. It displayed an HR = 0.74 (95%CI = 0.69–0.79; p = 6.2 × 10−21, Table 1 and Fig 2). The correlation with the top SNP in *BRCA1* mutation carriers was 0.88 and with the top SNP in *BRCA2* mutation carriers 0.69 (Table 2). In addition, 148 SNPs reached genome wide significance (p < 5 × 10−8) for the association with ovarian cancer risk, including the original GWAS hit rs3814113 (Fig 2). No evidence for heterogeneity in the associations for *BRCA1* and *BRCA2* mutation carriers was observed (Q-test, p-values >0.5, data not shown).

### Identifying Independent Signals for the Association of 9p22 and Ovarian Cancer in *BRCA1* and *BRCA2* Mutation Carriers

In *BRCA1* mutation carriers, no variant displayed evidence of an association at a p <10−4 after analyses conditioning on rs10124837 (S1A Fig). The association with rs3814113, the original GWAS hit, became non-significant (p = 0.2) when rs10124837 was included as covariate in the model (S1A Fig and Table 3). Similarly, in *BRCA2* mutation carriers no evidence of an association was observed for any variant after conditioning on rs62543585 (p >10−4 S1B Fig). Neither rs3814113 nor rs10124837 were significant at p<0.05 when rs62543585 was included as covariate in the model while the latter still displayed an association with p = 5x10^-3^ (S1B Fig and Table 3).

**Table 3:** Conditional associations for *BRCA1* and *BRCA2* top SNPs. **The table shows the HR estimate. 95% CI and p-value for the conditional analysis adjusting for the lead SNP in the univariate analysis for *BRCA1* (left hand side) or *BRCA2* mutation carriers (right had side).** SNPs correspond to: rs10124837, the strongest associated in *BRCA1*; rs62543583, the strongest associated in *BRCA2* mutation carriers; rs7046326, the strongest associated in *BRCA1/2* meta-analysis; rs3814113, was the strongest associated variant in the initial GWAS analysis. “HR”, hazard ratio; “CI”, confidence interval.

	*BRCA1* (adj. rs10124837)			*BRCA2* (adj. rs62543583)		
SNP	HR	95%CI	p-value	HR	95%CI	p-value
rs62543583	1.0	(0.76, 1.24)	0.99	0.67	(0.51, 0.88)	4.0x10^-3^
rs10124837	0.8	(0.72, 0.88)	9.0x10^-5^	0.99	(0.78, 1.27)	0.96
rs62543583				0.75	(0.61, 0.92)	5.0x10^-3^
rs3814113				0.87	(0.74, 1.03)	0.11
rs10124837	0.8	(0.72, 0.88)	1.5x10^-5^			
rs3814113	0.9	(0.86, 1.03)	0.20			

Taken together, these results indicate that in both *BRCA1* and *BRCA2* mutation carriers there is only one peak of association with ovarian cancer risk at 9p22.

#### Association of 9p22 and Ovarian Cancer in *BRCA1* and *BRCA2* Mutation Carriers

SNPs with a likelihood ratio relative to the most significant variant greater than 100 and having an *r*2< 0.1 with the index SNP were excluded from being potentially causative. In *BRCA1* mutation carriers, this identified eight highly correlated SNPs (r^2^>0.8), referred hereafter as the "*BRCA1* peak". These variants clustered in a 20kb region around the transcription start site of *BNC2* (positions: 16,847,520–16,891,647). The SNPs in this set displayed MAFs of 0.24–0.28 and imputation accuracy higher than 0.95 and two out of the eight were genotyped (Fig 1A and Table 1 and S4 Table).

In *BRCA2* mutation carriers, 100 variants could not be rejected from being potentially causal. The MAFs for these SNPs varied from 0.15 to 0.34 and had pairwise correlations with the index SNP of greater than 0.4 (Fig 1B, S4 Table). The quality of imputation was >0.95 for all except two variants (info = 0.68 and 0.46, S4 Table).

All except one (imputed SNP rs139555631) of the likely causal variants in *BRCA1* mutation carriers were included in the set marking the potentially causal variants defined in *BRCA2* mutation carriers. However, none of them were ranked within the top 60 associated variants in *BRCA2* carriers. The index SNP (rs10124837 in *BRCA1* mutation carriers was in linkage disequilibrium with the index SNP (rs62543583) in *BRCA2* mutation carriers r^2^ = 0.76, Fig 1, Table 2).

The original GWAS hit, rs3814113, was within the set of the strongest associated SNPs in *BRCA2* mutation carriers, but was rejected from being potentially causal in *BRCA1* mutation carriers.

In the *BRCA1/*2 meta-analysis, eleven SNPs were the set of potentially causal variants, which included the eight identified in *BRCA1* plus three only present in the *BRCA2* set. These eleven variants were highly correlated with the lead SNP of the meta-analysis rs7046326 (*r*2>0.8). Of note, the set excluded the original GWAS hit rs3814113 (Fig 2, S5 Table).

**Fig 3:**
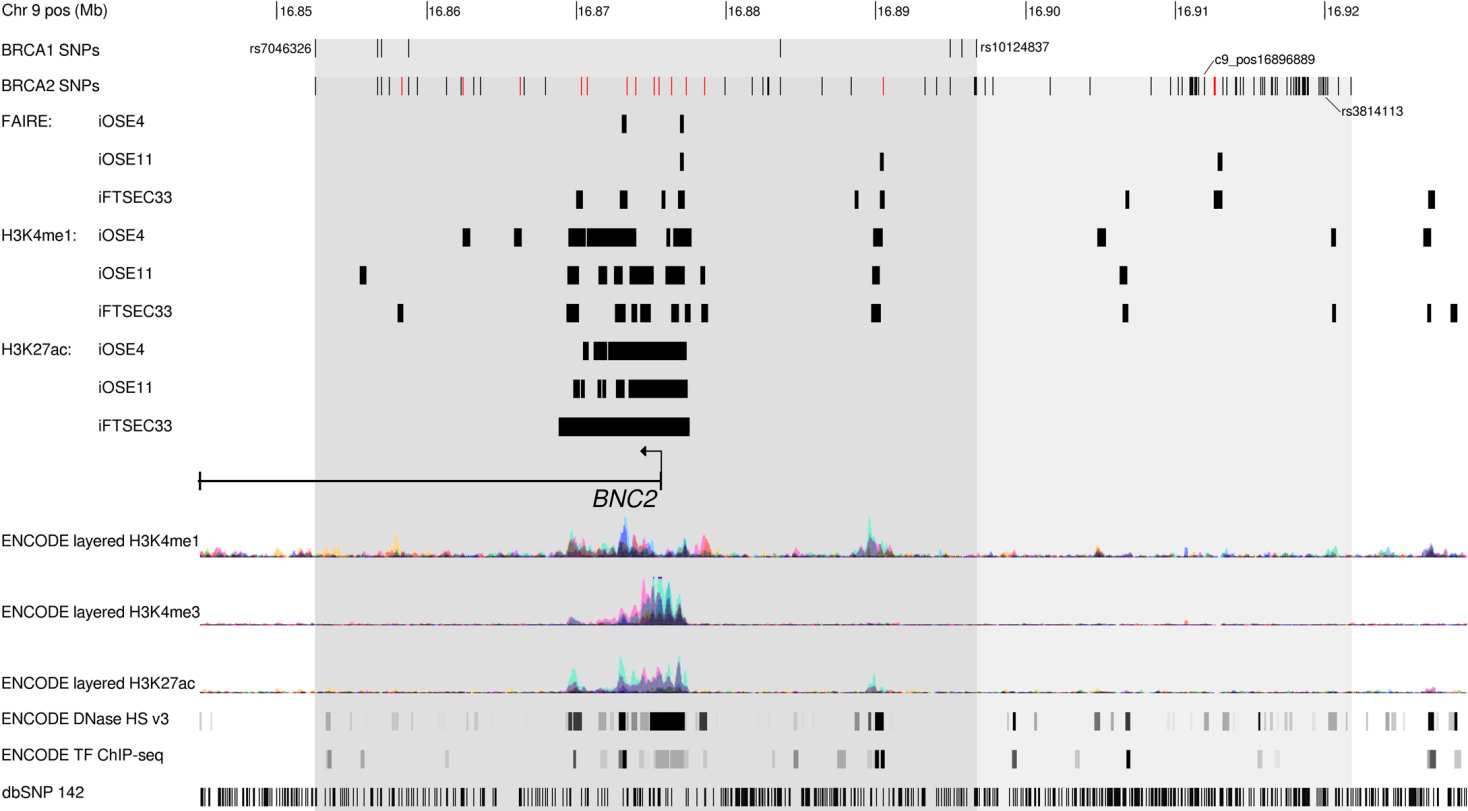
Genomic features surrounding the 9p22.2 locus. Illustration of the genomic region (chr9:16,839,835–16,924,468) encompassing peaks (shaded areas) containing candidate causal variants associated with ovarian cancer risk in *BRCA1* and *BRCA2* mutation carriers. Epigenomic data from Coetzee et al., (2015) [20] representing potential regulatory elements in ovarian cells (iOSE4 and iOSE11) and fallopian tube (FTSEC33) cells derived from formaldehyde assisted identification of regulatory elements sequencing (FAIRE-seq) and histone modification ChIP-seq are shown as black bars. Variants which overlap one of these features are coloured red. Data from the ENCODE project including histone modification ChIP-seq for three modifications (H3K4me1, H3K4me3, and H3K27ac) are shown as coloured histograms, as well as DNaseI hypersensitive site mapping and transcription factor ChIP-seq. The positions of all common SNPs from dbSNP build 142 are shown in the lowest track.

Intersection of variants exhibiting the strongest associations with genomic features derived from cultured ovarian and fallopian tube cells revealed several SNPs that may be functionally relevant in influencing risk. Fig 3 shows the location of the sets of SNPs associated with ovarian cancer risk in *BRCA1* and *BRCA2* mutation carriers relative to the *BNC2* gene. Several potentially functional variants are predicted, including SNPs that lie in regulatory regions identified by FAIRE- and ChIP-seq. For example, a cluster of eight SNPs from the *BRCA2* set of candidate causal variants lies within a ~10 kb region likely to carry regulatory activity encompassing the *BNC2* transcription start site. Multiple transcription factor motifs are altered by these variants (S6 Table). Although, no special features were observed for the variants in *BRCA1* or *BRCA1/2* meta-analysis (Fig 3), four of the eight candidate causal SNPs in *BRCA1* mutation carriers are expressed single nucleotide polymorphism (eSNP) for the *BNC2* gene in whole blood samples (Table 1, data extracted from GTex Portal http://www.gtexportal.org/home/).

## Discussion

In this study, we performed fine-scale mapping of the 9p22.2 locus using dense genotype data from the iCOGS array in *BRCA1* and *BRCA2* mutation carriers of European ancestry. We identified a set of variants that provided stronger evidence of association than the original GWAS hit.

In *BRCA1* mutation carriers, one independent set of eight highly correlated (*r*2>0.8) SNPs could not be excluded as being potentially causal for the reported association with ovarian cancer, designated the "*BRCA1* peak". The *BRCA1* peak covers positions 16847520 to 16891647, which lie within or up to 20 kb upstream *BNC2*. Of note, the original GWAS hit rs3814113 was excluded from the candidate causal variants in this peak.

For *BRCA2* mutation carriers, 100 correlated variants (*r*2>0.4) could not be excluded as potentially causal ("*BRCA2* peak"). The *BRCA2* peak spanned positions 16847520 to 16915021, which are up to 44 kb upstream of *BNC2* and more than 200kb upstream of *CNTLN*. The increased number of variants in this case is most likely due to reduced statistical power, as the number of *BRCA2* mutation carriers diagnosed with ovarian cancer was only one quarter of the number of affected *BRCA1* carriers. The candidate causal SNPs in the *BRCA1* peak were mostly contained within the *BRCA2* peak but the strongest associated SNP in *BRCA2* was excluded from the *BRCA1* peak. The current analysis was underpowered to investigate whether the association in *BRCA2* mutation carriers is driven by a different set of genetic variants.

Under the model of one shared causal variant explaining the association in both *BRCA1* and *BRCA2* mutation carriers, the meta-analysis would be expected to increase power for refining the set of potential causal variants. However, the combined analysis of *BRCA1* and *BRCA2* mutation carriers defined a set of eleven variants as potentially causal, which corresponded to the eleven strongest associated variants in *BRCA1*. This set excluded rs3814113 that was reported in the ovarian cancer GWAS [5]. The set of candidate causal variants included three additional SNPs that were confidently discarded on the basis of being less than 100 times likely to be causal relative to the strongest associated SNP in the analysis of *BRCA1* carriers only.

Important differences emerged when we compared the patterns of association in the fine-scale mapping of 9p22.2 between *BRCA1* mutation carriers and results for the most strongly associated SNPs in samples from the general population.

Fine-mapping results based on iCOGS data from the Ovarian Cancer Association Consortium indicate that SNP rs3814113 remains the most strongly associated SNP at the 9p22.2 region with serous ovarian cancer, the original GWAS hit (personal communication). Based on our results, this SNP can be confidently rejected from the set of possible causal variants in *BRCA1* mutation carriers, suggesting that the associations in *BRCA1* mutation carriers and in the general population may be driven by different causal variants at the 9p22.2 locus. These results may indicate differences in the underlying causal mechanisms explaining the ovarian cancer associations between *BRCA1* mutation carriers and the general population. In support of this possibility, differences in the association patterns with ovarian cancer between *BRCA1* and the general population have been reported before. The 4q32.3 locus is associated with ovarian cancer risk in *BRCA1* but not in *BRCA2* mutation carriers or the general population [11], while the opposite is true for the locus 17q11.2 [21]. However, clearer patterns will hopefully emerge once the fine mapping of the 9p22.2 region in samples from the general population is completed.

As both signals lie in close proximity to the *BNC2* gene, and some candidate causal SNPs are eSNPs for *BNC2* in whole blood, they may modulate the expression of *BNC2* through similar, or different, mechanisms. The possibility that the *BRCA1* association signal may differ from that in the general population adds extra complexity and reinforces the value of fine-scale mapping in different populations. These subtle differences in the patterns of associations depending on the underlying genetic landscape may be difficult to uncover by means other than fine-scale mapping, and thus strengthens the value of this approach for generating hypotheses about the functional basis of different sets of variants.

This study cannot exclude the possibility that the actual causal variants were not included in the set of genotyped or well-imputed variants. However, the iCOGs array included variants specifically for fine-scale mapping of the 9p22.2 locus based on data from the 1000 Genomes Project and therefore the region coverage is expected to be high. The relatively low number of ovarian cancer cases with tumor morphology information did not allow performing stratified analyses by ovarian cancer histological subtype. Studies of ovarian tumours in women with *BRCA1* or *BRCA*2 mutations have shown that *BRCA1* and *BRCA2* carriers predominantly develop serous disease [19,22]. Of the available data in CIMBA, 67% of all ovarian cancer tumours in our analyses were serous ovarian cancers. Our results are therefore more comparable with the associations for serous ovarian cancer in the general population. Larger studies will be required to assess whether the patterns of associations differ by ovarian cancer histological subtyped in *BRCA1*and *BRCA2* mutation carriers.

Having narrowed down the potential set of causal variants to only eight SNPs in *BRCA1* mutation carriers will assist functional studies to identify the gene/s targeted by these variants. *BNC2* is an obvious candidate gene, given that the putative causal variants are located in/around its transcription start site. Identifying more strongly associated variants with ovarian cancer in the 9p22.2 region relative to the initial GWAS hit in *BRCA1* and *BRCA2* mutation carriers will refine the cancer risks associated with this locus further. These novel variants can be included in polygenic risk scores for ovarian cancer and hence inform the identification of patients at greater risk of disease. The results may also help to deepen our understanding of the biology of ovarian cancer development in *BRCA1* and *BRCA2* mutation carriers, potentially leading to the development of more effective and personalized treatments.

## Supporting Information

S1 Fig Assessment for an independent signal for the association between SNPs in 9p22.2 and ovarian cancer risk in *BRCA1* and *BRCA2* mutation carriers. The colour code indicates the linkage disequilibrium with respect to the variant used for adjustment.
(TIFF)

S1 Table List of the local Institutional Review Boards that provided ethical approval for this study. (XLSX)

S2 Table Characteristics of study participants. (PDF)

S3 Table Association of SNPs with ovarian cancer risk in *BRCA1* and *BRCA2* mutation carriers (p<0.01). (XLSX)

S4 Table SNPs within 100 times likely of being causal for the association with ovarian cancer in *BRCA1* and *BRCA2* mutation carriers. 'T' corresponds to genotyped; 'Info' measures the accuracy of the imputation; 'Ref' and 'Eff' correspond to reference and effector allele, respectively; 'MAF' to minor allele frequency, 'HR' hazard ratio and 'CI' confidence interval. Bold cells correspond to the strongest associated SNP in the indicated dataset. Green and violet text indicates the set of potentially causal variant/s in *BRCA1* and *BRCA2* mutation carriers, respectively.
(PDF)

S5 Table SNPs within 100 times likely of being causal for the association with ovarian cancer in the meta-analysis of *BRCA1* and *BRCA2* mutation carriers. 'T' corresponds to genotyped; 'Ref' and 'Eff' correspond to reference and effector allele, respectively; 'MAF' to minimum allele frequency, 'HR' hazard ratio and 'CI' confidence interval. Bold cells correspond to the strongest associated SNP in the indicated dataset. Green, violet and orange text indicate those SNPs within 100 times likely of being the causal variant/s in *BRCA1* and *BRCA2* mutation carriers and their meta-analysis, respectively.
(PDF)

S6 Table Genomic features for selected SNPs associated with ovarian cancer risk in *BRCA2* mutation carriers. (XLSX)

S1 Text Full list of authors and affiliations. (DOCX)
