# Supplementary figures and images for: Fine-Scale Mapping at 9p22.2 Identifies Candidate Causal Variants That Modify Ovarian Cancer Risk in BRCA1 and BRCA2 Mutation Carriers
Source: PLoS One. 2016 Jul 27;11(7):e0158801. doi: 10.1371/journal.pone.0158801 (PMC4963094; doi:10.1371/journal.pone.0158801)

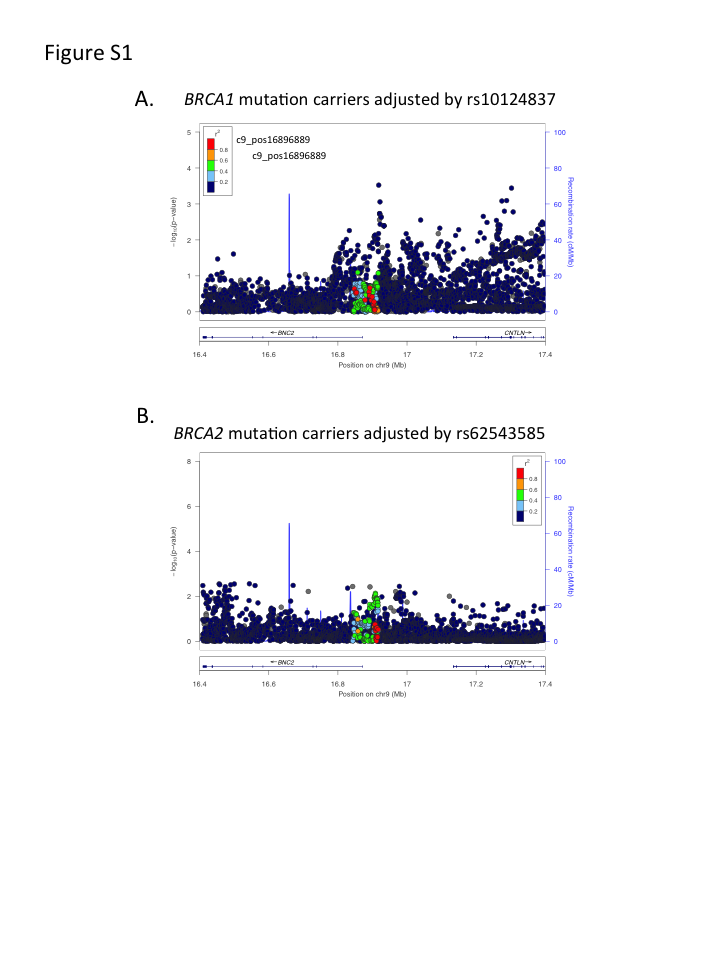

Supplement: S1 Fig — The colour code indicates the linkage disequilibrium with respect to the variant used for adjustment. (TIFF) [file pone.0158801.s001.tiff]
