# Supplementary material for: Fine-Scale Mapping at 9p22.2 Identifies Candidate Causal Variants That Modify Ovarian Cancer Risk in BRCA1 and BRCA2 Mutation Carriers
Source: PLoS One. 2016 Jul 27;11(7):e0158801. doi: 10.1371/journal.pone.0158801 (PMC4963094; doi:10.1371/journal.pone.0158801)
Supplement: S2 Table — (PDF) [file pone.0158801.s003.pdf]

Characteristic	*BRCA1*			*BRCA2*		
	Total	unaffected	ovarian cancer	Total	unaffected	ovarian cancer
N	15252	12790	2462	8211	7580	631
Person time (years)	684487	558166	126321	389820	353972	35848
Mean age (SD)	45(12)	44(12)	51(9)	47(13)	47(13)	57(10)
Age at censoring	N(%)	N(%)	N(%)	N(%)	N(%)	N(%)
[0,20)	89(0.6)	89(0.7)	0(0)	36(0.4)	36(0.5)	0(0)
[20,30)	1283(8.4)	1271(9.9)	12(0.5)	602(7.3)	597(7.9)	5(0.8)
[30,40)	4093(26.8)	3867(30.2)	226(9.2)	1761(21.4)	1747(23)	14(2.2)
[40,50)	5030(33)	4119(32.2)	911(37)	2543(31)	2411(31.8)	132(21.1)
[50,60)	3060(20.1)	2197(17.2)	863(35.1)	1850(22.5)	1619(21.3)	231(36.9)
[60,70)	1234(8.1)	870(6.8)	364(14.8)	987(12)	796(10.5)	191(30.5)
[70,80)	463(3.0)	390(3.0)	73(3.0)	432(5.3)	379(5.0)	53(8.5)
Year of birth						
[1900,1920)	67(0.4)	58(0.5)	9(0.4)	60(0.7)	48(0.6)	12(1.9)
[1920,1930)	326(2.1)	210(1.6)	116(4.7)	258(3.1)	196(2.6)	62(9.8)
[1930,1940)	939(6.2)	589(4.6)	350(14.2)	696(8.5)	533(7)	163(25.8)
[1940,1950)	2429(15.9)	1657(13)	772(31.4)	1506(18.3)	1267(16.7)	239(37.9)
[1950,1960)	3939(25.8)	3157(24.7)	782(31.8)	2155(26.2)	2029(26.8)	126(20)
[1960,1990)	7545(49.5)	7112(55.6)	433(17.6)	3536(43.1)	3507(46.3)	29(4.6)
Ovarian cancer morphology			N(%)			N(%)
Serous			795(68)			196(66)
Mucinous			15(1)			7(2)
Endometroid			128(11)			35(12)
Clear cell			14(1)			6(2)
Other			212(19)			52(18)

Table S2: Characteristics of study participants. Mean age corresponds to the age at censoring. SD corresponds to the standard deviation, N corresponds to the number of individuals.
