# Supplementary material for: Fine-Scale Mapping at 9p22.2 Identifies Candidate Causal Variants That Modify Ovarian Cancer Risk in BRCA1 and BRCA2 Mutation Carriers
Source: PLoS One. 2016 Jul 27;11(7):e0158801. doi: 10.1371/journal.pone.0158801 (PMC4963094; doi:10.1371/journal.pone.0158801)
Supplement: S4 Table — 'T' corresponds to genotyped; 'Info' measures the accuracy of the imputation; 'Ref' and 'Eff' correspond to reference and effector allele, respectively; 'MAF' to minor allele frequency, 'HR' hazard ratio and 'CI' confidence interval. Bold cells correspond to the strongest associated SNP in the indicated dataset. Green and violet text indicates the set of potentially causal variant/s in BRCA1 and BRCA2 mutation carriers, respectively. (PDF) [file pone.0158801.s005.pdf]

					BRCA1					*BRCA2*				
SNP	Position	Ref.	Eff.	T	Info	MAF	HR	95%CI	p-value	Info	MAF	HR	95%CI	p-value
**rs10124837**	16891647	T	C	N	0.98	0.24	0.73	(0.79,0.68)	**1.99e-16**	0.98	0.23	0.74	(0.85,0.64)	2.38e-05
rs7046326	16847520	G	A	Y	0.99	0.25	0.74	(0.69,0.79)	2.92e-16	1.00	0.24	0.74	(0.64,0.84)	1.33e-05
rs4961501	16851678	G	T	N	0.97	0.25	0.74	(0.79,0.69)	3.78e-16	0.98	0.24	0.74	(0.84,0.64)	1.27e-05
rs10810647	16853779	T	C	N	0.98	0.25	0.74	(0.79,0.69)	4.35e-16	0.98	0.24	0.73	(0.84,0.64)	1.11e-05
rs10962662	16889937	C	A	Y	1.00	0.24	0.74	(0.68,0.79)	5.7e-16	1.00	0.23	0.74	(0.64,0.85)	2.06e-05
rs7868157	16851977	A	C	N	0.97	0.24	0.74	(0.79,0.69)	6.45e-16	0.94	0.24	0.74	(0.85,0.64)	1.5e-05
rs139555631	16890684	C	CTATT	N	0.90	0.28	0.74	(0.79,0.68)	9.74e-16	0.90	0.27	0.77	(0.88,0.67)	0.00024
rs10756823	16878616	C	A	N	0.98	0.24	0.74	(0.69,0.79)	1.01e-15	0.98	0.23	0.74	(0.64,0.85)	1.82e-05
rs10962643	16857403	C	A	N	0.97	0.32	0.77	(0.72,0.82)	3.2e-14	0.97	0.31	0.74	(0.66,0.84)	4.95e-06
rs113780397	16907584	G	A	N	0.97	0.32	0.77	(0.72,0.83)	1.1e-13	0.97	0.32	0.74	(0.65,0.84)	2.32e-06
rs181552334	16907646	A	G	N	0.97	0.32	0.77	(0.72,0.83)	1.11e-13	0.97	0.32	0.74	(0.65,0.84)	2.31e-06
rs55689948	16908169	A	G	N	0.97	0.33	0.78	(0.73,0.83)	1.35e-13	0.98	0.32	0.75	(0.66,0.84)	4.69e-06
rs112442786	16908402	CTGGGAGGAAG	C	N	0.98	0.33	0.78	(0.73,0.83)	1.43e-13	0.98	0.32	0.75	(0.66,0.85)	4.81e-06
rs7045767	16910898	G	C	Y	0.99	0.33	0.78	(0.73,0.83)	1.65e-13	0.99	0.32	0.75	(0.66,0.85)	6.8e-06
rs7866677	16909051	T	C	N	0.98	0.33	0.78	(0.73,0.83)	1.71e-13	0.98	0.32	0.75	(0.66,0.85)	4.98e-06
rs10962684	16909333	G	A	N	0.98	0.33	0.78	(0.73,0.83)	1.91e-13	0.98	0.32	0.75	(0.66,0.85)	5.31e-06
rs35884621	16911411	T	TA	N	0.99	0.33	0.78	(0.73,0.83)	2.24e-13	0.99	0.32	0.75	(0.66,0.85)	6.2e-06
rs4366169	16911638	A	C	Y	1.00	0.33	0.78	(0.73,0.83)	2.6e-13	1.00	0.32	0.75	(0.66,0.85)	8.56e-06
rs10738467	16910677	C	T	Y	1.00	0.33	0.78	(0.73,0.83)	3.08e-13	1.00	0.32	0.75	(0.66,0.85)	5.93e-06
rs10738468	16910763	A	G	Y	1.00	0.33	0.78	(0.73,0.83)	3.8e-13	1.00	0.32	0.75	(0.66,0.85)	7.56e-06
rs4445329	16911757	G	A	Y	1.00	0.33	0.78	(0.73,0.83)	4.18e-13	1.00	0.32	0.75	(0.66,0.85)	7.6e-06
rs3814113	16915021	T	C	Y	1.00	0.33	0.78	(0.73,0.83)	5.15e-13	1.00	0.32	0.75	(0.66,0.85)	6.7e-06
rs10810671	16914835	A	C	Y	0.97	0.34	0.78	(0.73,0.83)	5.69e-13	1.00	0.32	0.75	(0.66,0.85)	6.21e-06
rs7032221	16914895	A	G	Y	1.00	0.33	0.78	(0.73,0.83)	5.77e-13	1.00	0.32	0.75	(0.66,0.85)	6.71e-06
rs7048397	16914703	T	C	N	0.99	0.33	0.78	(0.73,0.84)	5.92e-13	0.99	0.32	0.75	(0.66,0.85)	6.55e-06
rs6475094	16913616	G	A	N	0.98	0.33	0.78	(0.73,0.84)	6.23e-13	0.98	0.32	0.75	(0.66,0.85)	6.47e-06
rs10962693	16916693	C	T	N	0.87	0.31	0.77	(0.83,0.72)	6.23e-13	0.88	0.30	0.75	(0.86,0.66)	2.68e-05
rs6475093	16913514	G	C	N	0.98	0.33	0.78	(0.73,0.84)	6.39e-13	0.98	0.32	0.75	(0.66,0.85)	6.43e-06
rs6475092	16913473	G	A	N	0.98	0.33	0.78	(0.73,0.84)	6.4e-13	0.98	0.32	0.75	(0.66,0.85)	6.46e-06
rs4465052	16913043	A	G	N	0.98	0.33	0.78	(0.73,0.84)	6.75e-13	0.98	0.32	0.75	(0.66,0.85)	5.33e-06
rs4246134	16912988	C	T	N	0.98	0.33	0.78	(0.73,0.84)	6.91e-13	0.98	0.32	0.75	(0.66,0.85)	5.38e-06
rs35353585	16913171	T	TTTG	N	0.98	0.33	0.78	(0.73,0.84)	6.94e-13	0.98	0.32	0.75	(0.66,0.85)	6.41e-06
rs4631563	16913286	C	A	N	0.98	0.33	0.78	(0.73,0.84)	7.19e-13	0.98	0.32	0.75	(0.66,0.85)	6.51e-06
rs34131140	16913829	A	AT	N	0.98	0.33	0.78	(0.73,0.84)	7.29e-13	0.98	0.32	0.75	(0.66,0.85)	6.03e-06
**rs62543585**	16906889	T	C	Y	1.00	0.20	0.75	(0.69,0.81)	1.59e-12	1.00	0.19	0.69	(0.59,0.8)	**1.04e-06**
rs72713890	16906510	G	C	N	0.99	0.20	0.75	(0.7,0.81)	2.19e-12	0.99	0.19	0.69	(0.6,0.8)	1.39e-06
c9-pos16896307	16906307	A	G	Y	1.00	0.20	0.75	(0.7,0.81)	2.3e-12	1.00	0.19	0.69	(0.59,0.8)	1.2e-06
rs62543581	16906006	C	A	N	0.99	0.20	0.75	(0.7,0.81)	2.32e-12	0.99	0.19	0.69	(0.59,0.8)	1.14e-06
rs139327399	16906238	AC	A	N	0.99	0.20	0.75	(0.7,0.81)	2.43e-12	0.99	0.19	0.69	(0.59,0.8)	1.17e-06
c9-pos16896152	16906152	A	G	Y	1.00	0.20	0.75	(0.7,0.81)	2.47e-12	1.00	0.19	0.69	(0.59,0.8)	1.22e-06
c9-pos16896094	16906094	C	T	Y	1.00	0.20	0.75	(0.7,0.81)	2.52e-12	1.00	0.19	0.69	(0.59,0.8)	1.22e-06
c9-pos16895922	16905922	C	T	Y	1.00	0.20	0.75	(0.7,0.81)	2.55e-12	1.00	0.19	0.69	(0.59,0.8)	1.18e-06
rs34606230	16903362	A	T	N	0.98	0.20	0.75	(0.69,0.81)	2.74e-12	0.98	0.19	0.69	(0.6,0.81)	1.96e-06
rs62543579	16905170	T	C	N	0.99	0.20	0.75	(0.7,0.81)	2.86e-12	0.99	0.19	0.69	(0.59,0.8)	1.22e-06
rs10962670	16896588	C	T	N	0.98	0.20	0.75	(0.7,0.82)	3.77e-12	0.98	0.19	0.70	(0.6,0.81)	2.27e-06
c9-pos16894635	16904635	C	G	Y	1.00	0.20	0.76	(0.7,0.82)	5.12e-12	1.00	0.19	0.70	(0.6,0.81)	2.15e-06
c9-pos16889285	16899285	T	C	Y	0.99	0.20	0.76	(0.7,0.82)	7.7e-12	1.00	0.19	0.70	(0.6,0.81)	2.38e-06
rs10962664	16891561	T	C	N	0.98	0.20	0.76	(0.7,0.82)	8.31e-12	0.98	0.19	0.70	(0.6,0.81)	2.73e-06
rs10962665	16891590	A	G	N	0.98	0.20	0.76	(0.7,0.82)	8.37e-12	0.98	0.19	0.70	(0.6,0.81)	2.73e-06
rs80039758	16908383	C	G	N	0.98	0.20	0.76	(0.7,0.82)	1.15e-11	0.98	0.19	0.69	(0.6,0.8)	1.19e-06
rs12376998	16909002	A	T	N	0.98	0.20	0.76	(0.7,0.82)	1.38e-11	0.98	0.19	0.69	(0.6,0.8)	1.33e-06
9-16909474	16909474	G	A	N	0.97	0.20	0.76	(0.7,0.82)	1.98e-11	0.98	0.19	0.70	(0.6,0.81)	2.69e-06
9-16909473	16909473	T	TAC	N	0.97	0.20	0.76	(0.7,0.82)	1.99e-11	0.98	0.19	0.70	(0.6,0.81)	2.69e-06
9-16909475	16909475	T	TG	N	0.97	0.20	0.76	(0.7,0.82)	1.99e-11	0.98	0.19	0.70	(0.6,0.81)	2.69e-06
rs199782476	16909476	A	AGCT	N	0.97	0.20	0.76	(0.7,0.82)	1.99e-11	0.98	0.19	0.70	(0.6,0.81)	2.69e-06
c9-pos16900214	16910214	A	C	Y	1.00	0.20	0.76	(0.71,0.83)	2.21e-11	0.99	0.20	0.70	(0.6,0.81)	2.01e-06
rs10962691	16915105	C	G	Y	1.00	0.20	0.76	(0.71,0.83)	2.63e-11	1.00	0.20	0.70	(0.61,0.81)	2.48e-06
rs10810666	16911666	C	T	Y	1.00	0.20	0.76	(0.71,0.83)	2.73e-11	1.00	0.19	0.69	(0.6,0.81)	1.66e-06
rs62543619	16914716	G	A	N	0.98	0.20	0.76	(0.71,0.83)	2.78e-11	0.99	0.19	0.70	(0.6,0.81)	2.46e-06
rs10810665	16911400	G	A	Y	1.00	0.20	0.76	(0.71,0.83)	2.79e-11	1.00	0.19	0.69	(0.6,0.81)	1.66e-06
rs62543618	16914578	C	T	N	0.98	0.20	0.76	(0.71,0.83)	2.87e-11	0.99	0.19	0.70	(0.6,0.81)	2.21e-06
rs12377421	16913768	G	A	N	0.98	0.20	0.76	(0.71,0.83)	3.17e-11	0.98	0.19	0.70	(0.6,0.81)	1.78e-06
rs10756819	16858084	A	G	Y	1.00	0.34	0.80	(0.85,0.75)	3.24e-11	1.00	0.33	0.77	(0.87,0.68)	2.66e-05
rs12377389	16913557	C	T	N	0.98	0.20	0.76	(0.71,0.83)	3.65e-11	0.99	0.19	0.69	(0.6,0.8)	1.57e-06
rs10962692	16915874	G	C	N	0.92	0.20	0.76	(0.7,0.82)	3.99e-11	0.93	0.19	0.69	(0.6,0.81)	2.75e-06
rs10810668	16912435	G	C	Y	0.99	0.20	0.77	(0.71,0.83)	5.04e-11	1.00	0.19	0.70	(0.6,0.81)	2.04e-06
rs10810669	16912661	C	T	Y	0.98	0.20	0.77	(0.71,0.83)	5.93e-11	0.99	0.20	0.70	(0.6,0.81)	1.57e-06
rs10810670	16912663	C	T	Y	1.00	0.20	0.77	(0.71,0.83)	6.56e-11	1.00	0.19	0.69	(0.6,0.81)	1.55e-06
rs200306057	16892783	T	TTTTA	N	0.68	0.30	0.76	(0.82,0.7)	7.45e-11	0.68	0.30	0.69	(0.81,0.59)	2.97e-06
rs10962666	16892272	G	A	Y	1.00	0.20	0.77	(0.71,0.83)	1.09e-10	1.00	0.19	0.70	(0.6,0.81)	1.63e-06
rs34987069	16912842	CT	C	N	0.93	0.30	0.79	(0.74,0.85)	1.57e-10	0.94	0.29	0.73	(0.64,0.83)	2.54e-06
rs10962659	16883318	G	C	N	0.98	0.15	0.76	(0.69,0.83)	7.98e-10	0.98	0.14	0.71	(0.6,0.84)	7.68e-05
rs10962649	16873535	C	T	Y	1.00	0.15	0.76	(0.69,0.83)	8.98e-10	1.00	0.15	0.72	(0.61,0.85)	8.99e-05
rs12376099	16889023	G	A	N	0.98	0.15	0.76	(0.69,0.83)	9.05e-10	0.98	0.14	0.71	(0.6,0.84)	5.59e-05
rs10962658	16881346	G	A	N	0.98	0.15	0.76	(0.69,0.83)	9.36e-10	0.98	0.15	0.71	(0.6,0.84)	6.49e-05
c9-pos16875464	16885464	A	G	Y	1.00	0.15	0.76	(0.7,0.83)	9.54e-10	1.00	0.15	0.72	(0.61,0.85)	9.17e-05
rs10962653	16876736	T	C	N	0.99	0.15	0.76	(0.69,0.83)	1.02e-09	0.99	0.15	0.71	(0.6,0.84)	7.14e-05
rs62541926	16877423	T	C	N	0.99	0.15	0.76	(0.69,0.83)	1.05e-09	0.99	0.15	0.71	(0.6,0.84)	6.72e-05

rs140047332	16877723	GC	G	N	0.99	0.15	0.76	(0.69,0.83)	1.06e-09	0.99	0.15	0.71	(0.6,0.84)	6.56e-05
rs62541927	16888222	C	G	N	0.98	0.15	0.76	(0.69,0.83)	1.14e-09	0.98	0.14	0.71	(0.6,0.84)	7.99e-05
rs10962656	16877788	G	A	Y	1.00	0.15	0.76	(0.7,0.83)	1.2e-09	1.00	0.15	0.71	(0.6,0.83)	4.42e-05
rs11789875	16872323	G	A	N	0.98	0.15	0.76	(0.7,0.83)	1.52e-09	0.98	0.15	0.71	(0.6,0.84)	6.15e-05
rs10962652	16874878	A	G	N	0.97	0.15	0.76	(0.69,0.83)	2.19e-09	0.97	0.14	0.71	(0.6,0.84)	5.98e-05
rs62541923	16870501	C	A	N	0.98	0.15	0.76	(0.7,0.83)	2.24e-09	0.98	0.15	0.71	(0.6,0.84)	5.95e-05
rs200648906	16871360	C	CG	N	0.98	0.15	0.76	(0.7,0.83)	2.41e-09	0.98	0.15	0.71	(0.6,0.84)	6.93e-05
rs62541922	16870182	T	C	N	0.98	0.15	0.76	(0.7,0.83)	2.64e-09	0.98	0.15	0.71	(0.6,0.84)	6.55e-05
rs201753327	16906414	T	TA	N	0.96	0.25	0.80	(0.74,0.86)	3.22e-09	0.96	0.25	0.73	(0.64,0.84)	9.08e-06
rs10962679	16905441	C	T	N	0.98	0.26	0.80	(0.86,0.75)	3.37e-09	0.98	0.25	0.73	(0.84,0.64)	6.28e-06
rs10962642	16857292	G	T	N	0.96	0.16	0.77	(0.71,0.84)	6.74e-09	0.97	0.16	0.72	(0.61,0.85)	8.64e-05
rs11788047	16858569	G	A	N	0.98	0.16	0.78	(0.71,0.85)	1.61e-08	0.98	0.16	0.72	(0.61,0.84)	7.19e-05
rs62541877	16852453	G	A	N	0.97	0.16	0.78	(0.71,0.85)	2.04e-08	0.97	0.16	0.71	(0.6,0.83)	3.84e-05
rs62541878	16861205	A	T	N	0.98	0.16	0.78	(0.72,0.85)	2.13e-08	0.98	0.16	0.72	(0.62,0.85)	9.67e-05
rs62541879	16861508	A	G	N	0.98	0.16	0.78	(0.72,0.85)	2.14e-08	0.98	0.16	0.72	(0.62,0.85)	9.78e-05
rs12379687	16854367	G	T	Y	1.00	0.16	0.78	(0.72,0.85)	2.46e-08	1.00	0.16	0.71	(0.61,0.83)	3.57e-05
rs10962648	16868958	G	C	Y	1.00	0.16	0.78	(0.72,0.85)	2.48e-08	1.00	0.16	0.73	(0.62,0.85)	9.97e-05
rs10962647	16868380	T	G	Y	0.93	0.17	0.78	(0.72,0.85)	2.86e-08	0.99	0.16	0.73	(0.62,0.85)	0.000105
rs60043391	16856277	A	T	N	0.46	0.27	1.34	(1.21,1.48)	2.93e-08	0.48	0.27	1.48	(1.23,1.79)	5.25e-05
rs200868580	16862913	A	ATT	N	0.98	0.16	0.78	(0.85,0.72)	3.43e-08	0.98	0.16	0.73	(0.85,0.62)	0.000125
c9-pos16855291	16865291	G	A	Y	1.00	0.16	0.78	(0.72,0.85)	3.91e-08	1.00	0.16	0.73	(0.62,0.85)	0.000112
rs10690336	16853296	TAGTC	T	N	0.97	0.16	0.78	(0.85,0.72)	4.75e-08	0.97	0.16	0.72	(0.85,0.61)	7.53e-05
rs12379183	16865699	A	G	Y	0.99	0.23	0.82	(0.76,0.88)	2.07e-07	0.98	0.23	0.76	(0.66,0.87)	7.16e-05

Table S4: SNPs within 100 times likely of being causal for the association with ovarian cancer in *BRCA1* and *BRCA2* mutation carriers. This set was defined based on a likelihood ratio less or equal than 100 times relative to the most significant variant and r^2>0.1 with the top SNP. 'T' correspond to genotyped; 'Info' measures the accuracy of the imputation; 'Ref' and 'Eff' correspond to reference and effector allele, respetively; 'MAF' to minor allele frequency, 'HR' hazard ratio and 'CI' confidence interval. Bold cells correspond to the strongest associated SNP in the indicated dataset. Green and violet text indicate the set of potentially causal variant/s in *BRCA1* and *BRCA2* mutation carriers, respectively.
