# Supplementary material for: Fine-Scale Mapping at 9p22.2 Identifies Candidate Causal Variants That Modify Ovarian Cancer Risk in BRCA1 and BRCA2 Mutation Carriers
Source: PLoS One. 2016 Jul 27;11(7):e0158801. doi: 10.1371/journal.pone.0158801 (PMC4963094; doi:10.1371/journal.pone.0158801)
Supplement: S5 Table — 'T' corresponds to genotyped; 'Ref' and 'Eff' correspond to reference and effector allele, respectively; 'MAF' to minimum allele frequency, 'HR' hazard ratio and 'CI' confidence interval. Bold cells correspond to the strongest associated SNP in the indicated dataset. Green, violet and orange text indicate those SNPs within 100 times likely of being the causal variant/s in BRCA1 and BRCA2 mutation carriers and their meta-analysis, respectively. (PDF) [file pone.0158801.s006.pdf]

					BRCA1				*BRCA2*				Meta-analysis		
SNP	Position	Ref.	Eff.	T	MAF	HR	95%CI	p-value	MAF	HR	95%CI	p-value	HR	95%CI	p-value
**rs7046326**	16847520	G	A	Y	0.25	0.74	(0.69,0.79)	2.92e-16	0.24	0.74	(0.64,0.84)	1.33e-05	0.74	(0.69,0.79)	**6.21e-21**
**rs10124837**	16891647	T	C	N	0.24	0.73	(0.79,0.68)	**1.99e-16**	0.23	0.74	(0.85,0.64)	2.38e-05	0.73	(0.69,0.78)	7.54e-21
rs4961501	16851678	G	T	N	0.25	0.74	(0.79,0.69)	3.78e-16	0.24	0.74	(0.84,0.64)	1.27e-05	0.74	(0.69,0.79)	7.75e-21
rs10810647	16853779	T	C	N	0.25	0.74	(0.79,0.69)	4.35e-16	0.24	0.73	(0.84,0.64)	1.11e-05	0.74	(0.69,0.79)	7.94e-21
rs7868157	16851977	A	C	N	0.24	0.74	(0.79,0.69)	6.45e-16	0.24	0.74	(0.85,0.64)	1.5e-05	0.74	(0.69,0.79)	1.58e-20
rs10962662	16889937	C	A	Y	0.24	0.74	(0.68,0.79)	5.7e-16	0.23	0.74	(0.64,0.85)	2.06e-05	0.74	(0.69,0.79)	1.91e-20
rs10756823	16878616	C	A	N	0.24	0.74	(0.69,0.79)	1.01e-15	0.23	0.74	(0.64,0.85)	1.82e-05	0.74	(0.69,0.79)	3.06e-20
rs10962643	16857403	C	A	N	0.32	0.77	(0.72,0.82)	3.2e-14	0.31	0.74	(0.66,0.84)	4.95e-06	0.76	(0.72,0.81)	3.21e-19
rs139555631	16890684	C	CTATT	N	0.28	0.74	(0.79,0.68)	9.74e-16	0.27	0.77	(0.88,0.67)	0.00024	0.74	(0.7,0.79)	4.13e-19
rs113780397	16907584	G	A	N	0.32	0.77	(0.72,0.83)	1.1e-13	0.32	0.74	(0.65,0.84)	2.32e-06	0.77	(0.72,0.81)	6.17e-19
rs181552334	16907646	A	G	N	0.32	0.77	(0.72,0.83)	1.11e-13	0.32	0.74	(0.65,0.84)	2.31e-06	0.77	(0.72,0.81)	6.17e-19

Table S5: SNPs within 100 times likely of being causal for the association with ovarian cancer in the meta-analsysis of *BRCA1* and *BRCA2* mutation carriers. 'T' correspond to genotyped; 'Ref' and 'Eff' correspond to reference and effector allele, respetively; 'MAF' to minimun allele frequency, 'HR' hazard ratio and 'CI' confidence interval. Bold cells correspond to the strongest associated SNP in the indicated dataset. Green, violet and orange text indicate those SNPs within 100 times likely of being the causal variant/s in *BRCA1* and *BRCA2* mutation carriers and their meta-analysis, respectively.
