# Supplementary material for: Fine-Scale Mapping at 9p22.2 Identifies Candidate Causal Variants That Modify Ovarian Cancer Risk in BRCA1 and BRCA2 Mutation Carriers
Source: PLoS One. 2016 Jul 27;11(7):e0158801. doi: 10.1371/journal.pone.0158801 (PMC4963094; doi:10.1371/journal.pone.0158801)
Supplement: S1 Text — (DOCX) [file pone.0158801.s008.docx]

**Text S1**

**Full author list**

Elena Vigorito^1^, Karoline B. Kuchenbaecker^1^, Jonathan Beesley^2^, Julian Adlard^3^, Bjarni A. Agnarsson^4^, Irene L. Andrulis^5^, Banu K. Arun^6^, Laure Barjhoux^7^, Muriel Belotti^8^, Javier Benitez^9^, Andreas Berger^10^, Anders Bojesen^11^, Bernardo Bonanni^12^, Carole Brewer^13^, Trinidad Caldes^14^, Maria A. Caligo^15^, Ian Campbell^16^, Salina B. Chan^17^, Kathleen B.M. Claes^18^, David E. Cohn^19^, Jackie Cook^20^, Mary B. Daly^21^, Francesca Damiola^7^, Rosemarie Davidson^22^, Antoine de Pauw^8^, Capucine Delnatte^23^, Orland Diez ^24^, Susan M. Domchek^25^, Martine Dumont^26^, Katarzyna Durda ^27^, Bernd Dworniczak^28^, Douglas F. Easton^1^, Diana Eccles^29^, Christina Edwinsdotter Ardnor^30^, Ros Eeles^31^, Bent Ejlertsen^32^, Steve Ellis^33^, D. Gareth Evans^34^, Lidia Feliubadalo^35^, Florentia Fostira^36^, William D. Foulkes^37^, Eitan Friedman^38^, Debra Frost^33^, Pragna Gaddam^39^, Patricia A. Ganz^40^, Judy Garber^41^, Vanesa Garcia-Barberan^14^, Marion Gauthier-Villars^8^, Andrea Gehrig^42^, Anne-Marie Gerdes^43^, Sophie Giraud^44^, Andrew K. Godwin^45^, David E. Goldgar^46^, Christopher R. Hake^47^, Thomas V. O. Hansen^48^, Sue Healey^2^, Shirley Hodgson^49^, Frans B.L. Hogervorst^50^, Claude Houdayer^8^, Peter J. Hulick^51^, Evgeny N. Imyanitov^52^, Claudine Isaacs^53^, Louise Izatt^54^, Angel Izquierdo^55^, Lauren Jacobs^56^, Anna Jakubowska^27^, Ramunas Janavicius^57^, Katarzyna Jaworska-Bieniek^27^, Uffe Birk Jensen^58^, Esther M. John^59^, Joseph Vijai^60^, Beth Y. Karlan^61^, Karin Kast^62^, KConFab Investigators^63^, Sofia Khan^64^, Ava Kwong^65^, Yael Laitman ^66^, Jenny Lester^61^, Fabienne Lesueur^67^, Annelie Liljegren^68^, Jan Lubinski^27^, Phuong L. Mai^69^, Siranoush Manoukian^70^, Sylvie Mazoyer^7^, Alfons Meindl ^71^, Arjen R. Mensenkamp^72^, Marco Montagna^73^, Katherine L. Nathanson^25^, Susan L. Neuhausen^74^, Heli Nevanlinna^64^, Dieter Niederacher^75^, Edith Olah^76^, Olufunmilayo I. Olopade^77^, Kai-ren Ong^78^, Ana Osorio^79^, Sue Kyung Park^80^, Ylva Paulsson-Karlsson^81^, Inge Sokilde Pedersen^82^, Bernard Peissel^70^, Paolo Peterlongo^83^, Georg Pfeiler^84^, Catherine M. Phelan^85^, Marion Piedmonte^86^, Bruce Poppe^18^, Miquel Angel Pujana^87^, Paolo Radice^88^, Gad Rennert^89^, Gustavo C. Rodriguez^90^, Matti A. Rookus^91^, Eric A. Ross^92^, Rita Katharina Schmutzler^93^, Jacques Simard^26^, Christian F. Singer^94^, Thomas P. Slavin^95^, Penny Soucy^26^, Melissa Southey^96^, Doris Steinemann^97^, Dominique Stoppa-Lyonnet^8^, Grzegorz Sukiennicki^27^, Christian Sutter^98^, Csilla I. Szabo^99^, Muy-Kheng Tea^94^, Manuel R. Teixeira^100^, Soo-Hwang Teo^101^, Mary Beth Terry^102^, Mads Thomassen^103^, Maria Grazia Tibiletti^104^, Laima Tihomirova^105^, Silvia Tognazzo^73^, Elizabeth J. van Rensburg^106^, Liliana Varesco^107^, Raymonda Varon-Mateeva^108^, Athanassios Vratimos ^109^, Jeffrey N. Weitzel^95^, Lesley McGuffog^1^, Judy Kirk^110^, Amanda Ewart Toland^111^, Ute Hamann^112^, Noralane Lindor^113^, Susan J. Ramus^114^, Mark H. Greene^69^, Fergus J. Couch^115^, Kenneth Offit^116^, Paul D.P. Pharoah^117^, Georgia Chenevix-Trench^2^ and Antonis C. Antoniou^1^,^#^

**Full affiliation list**

^1^Centre for Cancer Genetic Epidemiology, Department of Public Health and Primary Care, University of Cambridge;^2^Department of Genetics, QIMR Berghofer Medical Research Institute, Herston Road, Brisbane, Australia 4029;^3^Yorkshire Regional Genetics Service, Chapel Allerton Hospital, Leeds, UK;^4^Department of Pathology, University Hospital (Landspitali) and University of Iceland School of Medicine, Hringbraut, 101 Reykjavik, Iceland;^5^Lunenfeld-Tanenbaum Research Institute, Mount Sinai Hospital, Toronto, Ontario M5G 1X5, Departments of Molecular Genetics and Laboratory Medicine and Pathobiology, University of Toronto, Ontario, Canada;^6^Department of Breast Medical Oncology and Clinical Cancer Genetics Program, University Of Texas MD Andersson Cancer Center, 1515 Pressler Street, CBP 5, Houston, TX, USA;^7^Bâtiment Cheney D, Centre Léon Bérard, 28 rue Laënnec, Lyon, France;^8^Service de Génétique Oncologique, Institut Curie, 26, rue d’Ulm, Paris Cedex 05, France;^9^(1) Human Genetics Group, Spanish National Cancer Centre (CNIO), Madrid, Spain;(2) Biomedical Network on Rare Diseases (CIBERER), Madrid, Spain;(3) Human Genotyping (CEGEN) Unit, Human Cancer Genetics Program, Spanish National Cancer Research Centre (CNIO), Madrid, Spain;^10^Dept of OB/GYN, Medical University of Vienna, Vienna, Austria;^11^Department of Clinical Genetics, Vejle Hospital, Kabbeltoft 25, Vejle, Denmark;^12^Division of Cancer Prevention and Genetics, Istituto Europeo di Oncologia (IEO), via Ripamonti 435, 20141 Milan, Italy;^13^Department of Clinical Genetics, Royal Devon & Exeter Hospital, Exeter, UK;^14^Molecular Oncology Laboratory, Hospital Clinico San Carlos, IdISSC (El Instituto de Investigación Sanitaria del Hospital Clínico San Carlos), Martin Lagos s/n, Madrid, Spain;^15^Section of Genetic Oncology, Dept. of Laboratory Medicine, University and University Hospital of Pisa, Pisa Italy;^16^Research Division, Peter MacCallum Cancer Centre, Locked Bag 1, A'Beckett Street, Melbourne, VIC 8006 AUSTRALIA;^171^600 Divisadero Street, C415, San Francisco, CA 94143 - 1714, USA;^18^Center for Medical Genetics, Ghent University, De Pintelaan 185, 9000 Gent, Belgium;^19^Jones Professor of Obstetrics and Gynecology, Ohio State University Columbus Cancer Council GYN Oncology 3651 Ridge Mill Drive,Columbus, OH 43026 US\n;^20^Sheffield Clinical Genetics Service, Sheffield Children’s Hospital, Sheffield, UK ;^21^Department of Clinical Oncology, Fox Chase Cancer Center, 333 Cottman Avenue, Philadelphia, PA 19111;^22^Department of Clinical Genetics, South Glasgow University Hospitals, Glasgow, UK;^23^Unité d'oncogénétique, ICO-Centre René Gauducheau, Boulevard Jacques Monod, 44805 Nantes Saint Herblain Cedex;^24^Oncogenetics Group. Vall d’Hebron University Hospital, Vall d’Hebron Institute of Oncology (VHIO), and Universitat Autònoma. Passeig Vall d'Hebron 119-129. Barcelona. Spain;^25^Department of Medicine, Abramson Cancer Center, Perelman School of Medicine at the University of Pennsylvania, 3400 Civic Center Boulevard, Philadelphia, PA 19104, USA;^26^Genomics Center, Centre Hospitalier Universitaire de Québec Research Center and Laval University, 2705 Laurier Boulevard, Quebec City (Quebec), Canada;^27^Department of Genetics and Pathology, Pomeranian Medical University, Polabska 4, Szczecin, Poland.;^28^Institute of Human Genetics, University of Münster, Münster, Germany;^29^University of Southampton Faculty of Medicine, Southampton University Hospitals NHS Trust, Southampton, UK;^30^Department of Radiation Sciences, Oncology, Umeå University, Umea, Sweden;^31^Oncogenetics Team, The Institute of Cancer Research and Royal Marsden NHS Foundation Trust, Sutton, UK;^32^Department of Oncology, Rigshospitalet, Copenhagen University Hospital, Blegdamsvej 9, DK-2100 Copenhagen, Denmark;^33^Centre for Cancer Genetic Epidemiology, Department of Public Health and Primary Care, University of Cambridge, Strangeways Research Laboratory, Worts Causeway, Cambridge, UK;^34^Genomic Medicine, Manchester Academic Health Sciences Centre, Institute of Human Development, Manchester University, Central Manchester University Hospitals NHS Foundation Trust, Manchester, UK.;^35^Molecular Diagnostic Unit, Hereditary Cancer Program, IDIBELL (Bellvitge Biomedical Research Institute),Catalan Institute of Oncology. Gran Via de l'Hospitalet, 199-203. 08908 L'Hospitalet. Barcelona, Spain;^36^Molecular Diagnostics Laboratory, (INRASTES) Institute of Nuclear and Radiological Sciences and Technology, National Centre for Scientific Research "Demokritos", Patriarchou Gregoriou & Neapoleos str., Aghia Paraskevi Attikis, Athens, GREECE;^37^Program in Cancer Genetics, Departments of Human Genetics and Oncology, McGill University, Montreal, Quebec, Canada;^38^The Susanne Levy Gertner Oncogenetics Unit, Institute of Human Genetics, Chaim Sheba Medical Center, Ramat Gan 52621, and Sackler Faculty of Medicine, Tel Aviv University, Ramat Aviv 69978, Israel;^39^Clinical Cancer Genetics Laboratory, Memorial Sloane Kettering Cancer Center, New York, NY, USA;^40^UCLA Schools of Medicine and Public Health, Division of Cancer Prevention & Control Research, Jonsson Comprehensive Cancer Center,650 Charles Young Drive South, Room A2-125 HS, Los Angeles, CA 90095-6900, USA\n;^41^Cancer Risk and Prevention Clinic, Dana-Farber Cancer Institute, 450 Brookline Avenue, Boston, MA, USA;^42^Centre of Familial Breast and Ovarian Cancer, Department of Medical Genetics, Institute of Human Genetics, University Würzburg, Germany;^43^Department of Clincial Genetics, Rigshospitalet 4062, Blegdamsvej 9, København Ø, Denmark;^44^Service de Génétique Moléculaire et Clinique, Hospices Civils de Lyon, Lyon cedex 04, France;^45^Department of Pathology and Laboratory Medicine, 3901 Rainbow Boulevard,4019 Wahl Hall East, MS 3040, University of Kansas Medical Center, Kansas City, Kansas, USA;^46^Department of Dermatology, University of Utah School of Medicine, 30 North 1900 East, SOM 4B454, Salt Lake City, UT 84132, USA;^47^City of Hope Clinical Cancer Genetics Community Research Network, 1500 East Duarte Road, Duarte, CA 91010;^48^Center for Genomic Medicine, Rigshospitalet, Copenhagen University Hospital, Blegdamsvej 9, DK-2100 Copenhagen, Denmark;^49^Medical Genetics Unit, St George's, University of London, UK;^50^Family Cancer Clinic, Netherlands Cancer Institute, P.O. Box 90203, 1000 BE, Amsterdam, The Netherlands;^51^Medical Director, Center for Medical Genetics, NorthShore University HealthSystem, Clinical Assisant Professor of Medicine, University of Chicago Pritzker School of Medicine, 1000 Central Street, Suite 620,Evanston, IL 60201,US\n;^52^N.N. Petrov Institute of Oncology, St.-Petersburg 197758, Russia;^53^Lombardi Comprehensive Cancer Center, Georgetown University, 3800 Reservoir Road NW, Washington, DC, USA;^54^Clinical Genetics, Guy’s and St. Thomas’ NHS Foundation Trust, London, UK;^55^Genetic Counseling Unit, Hereditary Cancer Program, IDIBGI (Institut d'Investigació Biomèdica de Girona), Catalan Institute of Oncology. Av. França s/n. 1707 Girona, Spain;^56^Clinical Genetics Research Laboratory, Dept. of Medicine, Memorial Sloan-Kettering Cancer Center, 1275 York Avenue, New York, NY, USA;^57^Vilnius University Hospital Santariskiu Clinics, Hematology, oncology and transfusion medicine center, Dept. of Molecular and Regenerative Medicine, Santariskiu st. ;State Research Institute Centre for Innovative medicine, Zygymantu st. 9, Vilnius, Lithuania;^58^Department of Clinical Genetics, Aarhus University Hospital, Brendstrupgaardsvej 21C, Aarhus N, Denmark;^59^Department of Epidemiology, Cancer Prevention Institute of California, 2201 Walnut Avenue, Suite 300, Fremont, CA 94538, USA;^60^Clinical Genetics Research Laboratory, Dept. of Medicine, Memorial Sloan-Kettering Cancer Center, 1275 York Avenue, New York, NY 10044, USA;^61^Women's Cancer Program at the Samuel Oschin Comprehensive Cancer Institute, Cedars-Sinai Medical Center, 8700 Beverly Boulevard, Suite 290W, Los Angeles, CA, USA;^62^Department of Gynaecology and Obstetrics, University Hospital Carl Gustav Carus, Technical University Dresden, Germany;^63^Kathleen Cuningham Consortium for Research into Familial Breast Cancer, Peter MacCallum Cancer Center, Melbourne, Australia;^64^Department of Obstetrics and Gynecology, University of Helsinki and Helsinki University Hospital, Biomedicum Helsinki, P.O. BOX 700 (Haartmaninkatu 8), 00029 HUS, Finland;^65^The Hong Kong Hereditary Breast Cancer Family Registry;Cancer Genetics Center, Hong Kong Sanatorium and Hospital, Hong Kong;Department of Surgery, The University of Hong Kong, Hong Kong ;^66^The Susanne Levy Gertner Oncogenetics Unit, Institute of Human Genetics, Chaim Sheba Medical Center, Ramat Gan 52621, Israel;^67^Genetic Epidemiology of Cancer team, Inserm U900, Institut Curie, Mines ParisTech, 26 rue d'Ulm, 75248 Paris cedex 05, France;^68^Department of Oncology, Karolinska University Hospital, Stockholm, Sweden;^69^Clinical Genetics Branch,DCEG, NCI, NIH, 9609 Medical Center Drive, Room 6E-454, Bethesda, MD, USA;^70^Unit of Medical Genetics, Department of Preventive and Predictive Medicine, Fondazione IRCCS (Istituto Di Ricovero e Cura a Carattere Scientifico ) Istituto Nazionale Tumori (INT), Via Giacomo Venezian 1, 20133 Milan, Italy;^71^Department of Gynaecology and Obstetrics, Division of Tumor Genetics, Klinikum rechts der Isar, Technical University Munich, Germany;^72^Department of Human Genetics, Radboud university medical centre, P.O. Box 9101, 6500 HB Nijmegen, The Netherlands;^73^Immunology and Molecular Oncology Unit, Veneto Institute of Oncology IOV - IRCCS, Via Gattamelata 64, Padua, Italy;^74^Department of Population Sciences, Beckman Research Institute of City of Hope, Duarte, CA USA;^75^Department of Gynaecology and Obstetrics, University Hospital Düsseldorf, Heinrich-Heine University Düsseldorf, Germany;^76^Department of Molecular Genetics, National Institute of Oncology, Budapest, Hungary;^775^841 South Maryland Avenue, MC 2115 Chicago, IL;^78^West Midlands Regional Genetics Service, Birmingham Women’s Hospital Healthcare NHS Trust, Edgbaston, Birmingham, UK ;^79^(1) Human Genetics Group, Spanish National Cancer Centre (CNIO), Madrid, Spain;(2) Biomedical Network on Rare Diseases (CIBERER), Madrid, Spain.;^80^Department of Preventive Medicine, Seoul National University College of Medicine, Department of Biomedical Science, Seoul National University Graduate School, and Cancer Research Institute, Seoul National University, 103 Daehak-ro, Jongno-gu, Seoul 110-799, Korea ;^81^Department of Immunology, Genetics and Pathology, Uppsala University, SE-751 85 Uppsala, Sweden;^82^Section of Molecular Diagnostics, Department of Biochemistry, Aalborg University Hospital, Reberbansgade 15, Aalborg, Denmark;^83^IFOM, The FIRC (Italian Foundation for Cancer Research) Institute of Molecular Oncology, c/o IFOM-IEO campus, via Adamello 16 , 20139 Milan, Italy.;^84^Medical University of Vienna, Währinger Gürtel 18-20, 1090 Vienna, Austria;^85^Department of Cancer Epidemiology, Moffitt Cancer Center, Tampa, Florida, USA;^86^NRG Oncology, Statistics and Data Management Center, Roswell Park Cancer Institute, Elm St & Carlton St, Buffalo, NY 14263, USA;^87^Translational Research Laboratory, IDIBELL (Bellvitge Biomedical Research Institute),Catalan Institute of Oncology, Barcelona, Spain;^88^Unit of Molecular Bases of Genetic Risk and Genetic Testing, Department of Preventive and Predicted Medicine, Fondazione IRCCS (Istituto Di Ricovero e Cura a Carattere Scientifico ) Istituto Nazionale Tumori (INT), c/o Amaedeolab, via GA Amadeo 42, 20133 Milan, Italy ;^89^Clalit National Israeli Cancer Control Center and Department of Community Medicine and Epidemiology, Carmel Medical Center and B. Rappaport Faculty of Medicine, 7 Michal St., Haifa 34362, Israel;^90^Division of Gynecologic Oncology, NorthShore University HealthSystem, Clinical Professor, Univ of Chicago, 2650 Ridge Avenue Suite 1507 Walgreens, Evanston, IL 60201, US;^91^Department of Epidemiology. Netherlands Cancer Institute, P.O. Box 90203, 1000 BE, Amsterdam, The Netherlands;^92^Biostatistics and Bioinformatics Facility, Fox Chase Cancer Center, 333 Cottman Avenue, Philadelphia, PA 19111, USA;^93^(1) Center for Hereditary Breast and Ovarian Cancer, Medical Faculty, University Hospital Cologne, Germany;(2) Center for Integrated Oncology (CIO), Medical Faculty, University Hospital Cologne, Germany;(3) Center for Molecular Medicine Cologne (CMMC), University of Cologne, Germany (4) on behalf of the German Consortium of Hereditary Breast and Ovarian Cancer (GC-HBOC);^94^Dept of OB/GYN, Medical University of Vienna, Vienna, Austria, Waehringer Guertel 18-20, A 1090 Vienna, Austria;^95^Clinical Cancer Genetics, City of Hope, 1500 East Duarte Road, Duarte, California 91010 USA;^96^Genetic Epidemiology Laboratory, Department of Pathology, University of Melbourne, Parkville, Victoria, Australia;^97^Institute of Cell and Molecular Pathology, Hannover Medical School, Hannover, Germany;^98^Institute of Human Genetics, Department of Human Genetics, University Hospital Heidelberg, Germany;^99^National Human Genome Research Institute,National Institutes of Health Building 50, Room 5312, 50 South Drive, MSC 004, Bethesda, MD, USA, Bethesda, MD 20892-8004;^100^Department of Genetics, Portuguese Oncology Institute, Rua Dr. António Bernardino de Almeida, 4200-072 Porto, Portugal;^101^Cancer Research Initiatives Foundation, Sime Darby Medical Centre, 1 Jalan SS12/1A, Subang Jaya, 47500 Malaysia and University Malaya Cancer Research Institute, University Malaya, 50603 Kuala Lumpur, Malaysia;^102^Department of Epidemiology, Columbia University, New York, NY, USA;^103^Department of Clinical Genetics, Odense University Hospital, Sonder Boulevard 29, Odense C, Denmark;^104^UO Anatomia Patologica, Ospedale di Circolo-Università dell'Insubria, Via O.Rossi 9, 21100 Varese, Italy;^105^Latvian Biomedical Research and Study Centre. Ratsupites str 1, Riga, Latvia;^106^Cancer Genetics Laboratory, Department of Genetics, University of Pretoria,Private Bag X323, Arcadia 0007, South Africa;^107^Unit of Hereditary Cancer, Department of Epidemiology, Prevention and Special Functions, IRCCS (Istituto Di Ricovero e Cura a Carattere Scientifico ) AOU San Martino - IST Istituto Nazionale per la Ricerca sul Cancro, largo Rosanna Benzi 10, 16132 Genoa, Italy;^108^Institute of Human Genetics, Campus Virchov Klinikum, Charite Berlin, Germany;^109^Molecular Diagnostics Laboratory, (INRASTES) Institute of Nuclear and Radiological Sciences and Technology, National Centre for Scientific Research "Demokritos"\nPatriarchou Gregoriou & Neapoleos str., Aghia Paraskevi Attikis\n Athens, GREECE;^110^Westmead Hospital, Familial Cancer Service, Hawkesbury Road, P.O. Box 533, Wentworthville, NSW 2145, Australia, on behalf of Australia New Zealand Gynaecological Oncology Group (ANZGOG);^111^Divison of Human Cancer Genetics, Departments of Internal Medicine and Molecular Virology, Immunology and Medical Genetics, Comprehensive Cancer Center, The Ohio State University,998 Biomedical Research Tower, Columbus, OH, USA;^112^Molecular Genetics of Breast Cancer, German Cancer Research Center (DKFZ), Im Neuenheimer Feld 580, 69120 Heidelberg, Germany;^113^Department of Health Sciences Research, Mayo Clinic, 13400 E. Scottsdale Blvd., Scottsdale, AZ, USA;^114^Department of Preventive Medicine, Keck School of Medicine, University of Southern California, California, USA;^115^Department of Laboratory Medicine and Pathology, and Health Sciences Research, Mayo Clinic, 200 First Street SW, Rochester, Minnesota, USA;^116^Clinical Genetics Research Laboratory, Dept. of Medicine, Cancer Biology and Genetics, Memorial Sloan-Kettering Cancer Center, 1275 York Avenue, New York, NY 10044, USA and ^117^Department of Oncology, University of Cambridge, Cambridge, UK.
